# The impact of visuomotor skills on two pen-and-paper tests of sustained attention (d2-R, FAIR)

**DOI:** 10.1007/s00426-025-02198-x

**Published:** 2025-11-28

**Authors:** Peter Wühr, Bianca Wühr, Gerhard Rinkenauer

**Affiliations:** 1 TU Dortmund University, Dortmund, Germany; 2 University of Applied Sciences and Arts, Dortmund, Germany; 3 Leibniz Research Centre for Working Environment and Human Factors, Dortmund, Germany

## Abstract

We investigated the impact of motor skills on performance in two pen-and-paper tests of selective attention, the d2-R and the FAIR-2. Both tests require a visual search for target stimuli presented in a random order with distractor stimuli. In the d2-R, participants have to mark each target with a short stroke. In the FAIR-2, participants are required to draw a continuous line under each stimulus series that remains below the distractors, whereas targets are marked by drawing an upward spike into the target. The purpose of continuous marking in the FAIR-2 is to make the direction and order of participants’ visual search apparent to the experimenter. We tested 120 participants with the d2-R, the FAIR-2, and five subtests (aiming, pegboard, steadiness, tapping, line-tracking) of the MLS, a test battery of fine motor skills. Our results revealed similarities and differences between the motor demands of the two tests. Performance on both tests correlated with tapping (hits) and line-tracking (time). In contrast, only FAIR-2 performance correlated with pegboard (time) and steadiness (errors). Regression analyses showed that MLS performance explained a significant amount of variance in FAIR-2 performance, but not in d2-R performance. When directly compared, the differences in explained variance were not significant, however. Hence, the results suggest that the sum of motor demands in the d2-R and the FAIR-2 are comparable, although there are some differences in specific motor requirements.

**Supplementary Information:**

The online version contains supplementary material available at 10.1007/s00426-025-02198-x.

## Introduction

The ability to work in a concentrated manner is an important prerequisite for good performance in many areas such as school, academics, occupations, and sports (e.g., Bates & Lemay, 2004; Gallen et al., 2023); Kittler et al., 2022). The ability to concentrate on a task is closely related to the concept of selective or focused attention: Both concepts refer to the ability of efficiently processing relevant information, while effectively ignoring irrelevant information, for a particular period of time (e.g., Moran, 2012; Wühr & Wühr, 2023). Several psychometric tests have been developed for measuring the individual ability to concentrate—sometimes called tests of “sustained” attention—like the test d2 (e.g., Brickenkamp & Zillmer, 1998) and the FAIR^1^ test (e.g., Moosbrugger & Oehlschlägel, 1996). These pen-and-paper tests consist of one or more sheets of paper on which a series of visual stimuli is presented in rows. The participants’ task is to search for a particular set of target stimuli that are intermingled with irrelevant distractor stimuli, and to mark each target with a pen. Notably, targets have to be marked in different ways in the d2 and in the FAIR test. In the d2, participants are supposed to cross out each target with a short stroke. In contrast, in the FAIR, participants are supposed to draw a continuous line (from left to right) below each row of stimuli, and to mark each target with an upward spike into the target. Hence, the motor processes required for marking targets are quite different in these two tasks, and therefore we assume that the participants’ motor skills may influence performance in the two tests to a different degree. In the present study, we therefore investigate, and compare, how motor skills affect performance in the tests d2 and FAIR.

## The test d2

The test d2 was developed by Brickenkamp (1962) as a short test for measuring the ability of professional drivers. A major revision of the d2 test, the d2-R, was published in 2010 (Brickenkamp et al., 2010). Both the d2 and the d2-R are available in different languages including English (Brickenkamp & Zillmer, 1998), French (Brickenkamp et al., 2014), Spanish (cf. Rivera et al., 2017), and Japanese (cf. Yato et al., 2019).

The d2 consists of a single sheet of paper with 14 rows of stimuli (e.g., Brickenkamp & Zillmer, 1998). The stimuli are different combinations of the letters “d” or “p” with one to four vertical dashes that are printed above and/or below the letter. The three possible combinations of the letter “d” with two dashes are target stimuli. Different combinations of the letter “d” with one, three or four dashes, and combinations of the letter “p” with two dashes, form the set of distractor stimuli. The participants’ task is to search for targets through each row from left to right, and to mark each target with a short stroke through the target. Depending on the presumed ability level, participants are given either 15–20 s for searching each row.

Different test scores have been used for describing participants’ performance in the d2 test. Until the ninth edition of the test (Brickenkamp, 2002), the most important measure was the sum of correctly inspected items (called “GZ-F”). This score was computed by summing up all stimuli (targets and distractors) to the left of the last marked stimuli across all rows (GZ), and subtracting the sum of errors (F) from the first result. However, the score GZ-F is susceptible to strategic behaviors of the participants. For example, when participants skipped groups of stimuli while searching through a row, then GZ would increase more than F, producing a higher GZ-F score (e.g., Oehlschlägel & Moosbrugger, 1991). Therefore, starting with the ninth edition of the test d2, it was recommended to compute the sum of correctly marked targets (called “KL”) as a primary measure of performance. In addition, the percentage of errors (F%) should also be determined as a secondary measure.

### The FAIR test

Moosbrugger and Oehlschlägel (1996) developed the FAIR test as a methodologically improved alternative to the d2 test. In 2011, Moosbrugger and Oehlschlägel published a revised version of the FAIR, called FAIR-2, with updated norms. The original FAIR test has been available in German, English, and French, whereas the FAIR-2 is only available in German and in Korean (Moosbrugger et al., 2002).

The FAIR and the FAIR-2 come as a booklet with 8 pages. On the title page, there is space for filling in the date of testing and some demographic information of the participant. Pages 2 and 3 contain a detailed instruction for the test. Pages 4–5 and 6–7 contain a test sheet on the left page (i.e., on page 4 and 6), whereas fields for filling in sums and scores are printed on the right page (i.e. on page 5 and 7). Each test page contains 16 rows with 20 stimuli each. The stimuli consist of a larger circle that includes either a second, smaller, circle or a square, which in turn contains either two or three dots. The combination of two (inner) shapes (circle, square) and two amounts of dots (two, three) gives four different stimuli. Two stimuli are target stimuli, whereas the remaining two stimuli are distractor stimuli.

The participants’ task in the FAIR test is to search each row from left to right for targets. While searching, participants are instructed to draw a continuous line below the currently inspected stimulus row, and to mark each target with an upward spike that hits the target. Moosbrugger and Oehlschlägel (1996) call this requirement the “principle of complete marking”, but we will use the term “continuous marking” instead. Participants are given three minutes search time for each of the two test pages.

Three test scores (L, Q, K) are computed for describing performance in the FAIR and the FAIR-2. The score “L” is a measure of the speed of processing. L is the sum of correctly inspected stimuli (i.e., hits + correct rejections) minus two times the sum of errors (false alarms + misses). The score “Q” is a measure of the accuracy of processing. Q is the proportion of correctly processed items (hits + correct rejections) relative to all inspected items. Finally, the score “K” is a measure of the continuity or stability of performance. K is the product of L and Q (i.e., L × Q).

For the purpose of the present study, the most important difference between d2 and FAIR consists in the different motor demands that are imposed by different instructions for marking targets (and not marking distractors). Instructions for the d2 simply require participants to selectively cross out targets. In contrast, instructions for the FAIR/FAIR-2 require participants to draw a continuous line that remains below the distractors, and to mark each target by drawing an upward spike to the target. The requirement of continuous marking in the FAIR test is supposed to have several advantages as compared to the d2 (Moosbrugger & Oehlschlägel, 1996). First, it is supposed to force participants in the FAIR to search each stimulus row (a) only once, (b) without interruption (i.e. without skipping stimuli), and (c) in the instructed direction (i.e., from left to right). Any violation of these instructions could be seen in the line, and would be counted as a “line error”^2^. In contrast, the experimenter cannot determine from a test page of the d2 whether (a) participants had inspected a particular passage more than once, (b) whether a particular passage was skipped, and (c) whether participants obeyed to the instructed search direction.

### Convergent and discriminant validity of d2-R and FAIR-2

Both the d2 and the FAIR test are supposed to measure the same theoretical construct—the ability to concentrate, or the capacity of focused/selective attention—with similar tasks. In both tests, the task is a visual-search task, or, to be more precise, a so-called conjunction-search task (cf. Chan & Hayward, 2013; Treisman, 2009, for reviews). Therefore, one should expect high correlations between performance scores of the same participants in the two tests. In fact, several studies observed correlations of intermediate size (*r* ≈.50) between performance scores in the d2 and the FAIR. For example, Moosbrugger and Oehlschlägel (2011) report the results of a study by Schäfer and Moosbrugger (1993), who observed a correlation of *r* =.49 between the d2 score GZ-F and the FAIR score L in a sample of 601 participants. Hallwachs (1994) observed a very similar correlation of *r* =.49 between GZ-F and L. In a more recent study, Wühr and Ansorge (2020) found a lower correlation of *r* =.38 between the d2-R score KL and the FAIR-2 score L.

While attempting to measure the same construct, the two tests impose different motor requirements on the participants. We assume that the continuous-marking requirement in the FAIR test creates both different, and higher, motor demands for the participants than the discrete-marking requirement in the d2 test. Therefore, we would expect that performance in the FAIR test correlates more strongly with measures of motor skill than performance in the d2 test. Unfortunately, studies of correlations between search performance and measures of motor skill are only available for the d2 test, and not for the FAIR test. In studies of the d2, two measures of motor skill were considered: reaction time (RT) to simple visual stimuli, and the number of marked circles that were presented at target locations on a sheet of paper, but without distractors. Some studies with student participants revealed nonsignificant correlations between the RT and GZ-F (*r* = −.07, Schmidt-Atzert et al., 2006), whereas others observed significant (negative) correlations between RT and KL (*r* = −.20, Schwalbach, in prep., cited in Brickenkamp et al., 2010). When, however, motor skill was measured as the number of marked circles, significant correlations occurred between this measure and GZ (*r* =.27, Enders, 2004; *r* =.23, Schmidt-Atzert & Bühner, 1997), and also between the number of marked circles and KL (*r* =.24, Enders, 2004).

### The present study

In the present study, we investigate and compare the impact of motor skills on performance in two pen-and-paper tests of sustained attention, the d2-R and the FAIR-2. In particular, we investigate the possibility that different instructions concerning the way of marking targets (and nontargets) in the two tests impose different motor demands when doing the test. We used the “motor performance series” (Motorische Leistungsserie, MLS, Neuwirth & Benesch, 2004, 2012; see also Sülzenbrück et al., 2010) for assessing the fine motor skills of our participants. The MLS was developed based on Fleishman’s factor analysis studies (e.g. Fleishman, 1972) in order to operationalize specific fine motor skills. These factors are considered to be individual abilities that are partly attributed to biological inheritance and partly to previous experience and practice (see, for example, Anderson et al., 2021, or Ben-Zaken et al., 2019). The MLS consists of five tasks, or subtests, that measure different aspects of fine motor skill. The “steadiness” task provides a measure of hand steadiness, that is, the ability to take a particular arm-hand posture, and to maintain this posture over a longer period of time. The “line-tracking” task measures the speed and accuracy of continuous arm-hand movements. The “aiming” task garners the speed and precision of discrete goal-directed arm-hand movements. The “tapping” task measures the speed of simple, repetitive hand movements. Finally, the “pegboard” task investigates eye-hand coordination, and assesses the precision of finger movements.

The present study has three aims. First, we investigate whether motor skill has a significant impact on performance in two different pen-and-paper tests of sustained attention—the d2-R and the FAIR-2. The results of previous research already showed that motor skill can affect performance in the d2 test. Corresponding research for the FAIR-2 is lacking. Second, we investigate whether different motor skills have different effects on performance in the d2-R and the FAIR-2. We used multiple regression analysis for addressing this question, and compared the predictive power of performance in the five subtests of the MLS on performance in the d2-R to the predictive power of performance in the five subtests of the MSL on performance in the FAIR-2. We expected that motor skill would have a higher impact on performance in the FAIR-2 than in the d2-R because motor demands imposed by the FAIR-2 appear higher than those imposed by the d2-R. Third, we seek to better understand the particular motor demands that are imposed by the d2-R and the FAIR-2. The d2-R requires discrete, goal-directed movements for marking targets, and therefore performance in the d2-R could correlate more strongly than performance in the FAIR-2 with performance in the MLS tasks “aiming” and “tapping”. In contrast, the FAIR-2 requires continuous, goal-directed movements for marking targets, and therefore performance in the FAIR-2 could correlate more strongly than performance in the d2-R with performance in the MLS task “line-tracking”. These predictions, however, were not logically delineated from a theory, but rather resulted from our informal evaluation of the motor demands in the two tests of sustained attention.

## Method

### Openness and transparency

The local Ethics Committee of the TU Dortmund University approved the experimental protocol for this study (GEKTUDO_2021-24) in 2021. Moreover, we preregistered this study at OSF on November 3, 2021 (10.17605/OSF.IO/6A4W3). We report how we determined our sample size, data exclusion, all manipulations, and all measures in the study. Note that the present research did not produce digital raw data files. Raw data consisted of test sheets completed by participants, and in test protocols where experimenters noted measures from the MLS. These test sheets and protocols will not be published, but Excel sheets with individual tests scores can be obtained from the authors upon request.

### Participants

We used a calculator provided by Hemmerich (2019) for conducting an a-priori power analysis to determine the sample size of linear multiple regression models with five predictors (and two-tailed testing). Our goal was to obtain a power of 1-β = 0.95 for detecting an R² = 0.16 at the standard 0.05 alpha-error probability. The power analysis revealed target sample size of 110 participants for our study. Since we had 12 different orders of conditions, we decided to collect data from a multiple of 12, and increased the target sample size to 120 participants. Moreover, during testing, we observed that participants with long fingernails had difficulties in several tasks, particularly in the pegboard task, resulting in high error rates and long completion times. Consistent with our preregistration, we replaced eight participants with new participants.

We collected data from a total of 131 participants, mostly students at TU Dortmund University. Two data sets were lost due to equipment malfunction. The remaining sample of 129 participants had an average age of 23.02 years (*SD* = 4.42), and consisted of 98 females and 31 males. According to self-report, all participants had normal or corrected-to-normal vision, and no history of neurological impairment. The majority of participants (*N* = 115) classified themselves as right-handers, whereas the remaining participants (*N* = 14) classified themselves as left-handers. Participants gave their informed consent prior to participation and received either course credit or a payment of 12 Euro in exchange.

From the sample of 129 participants, eight participants were excluded because they had long fingernails, which impaired performance in several tasks, particularly in the pegboard task. The average time in the pegboard task of the excluded participants was 105 s, compared to a mean of 48 s of the remaining participants. Moreover, one additional participants was excluded because his age (49) exceeded the age range (18–40) that had been set in the preregistration. Therefore, the final sample included 120 participants.

### Apparatus and stimuli

**Fig. 1:**
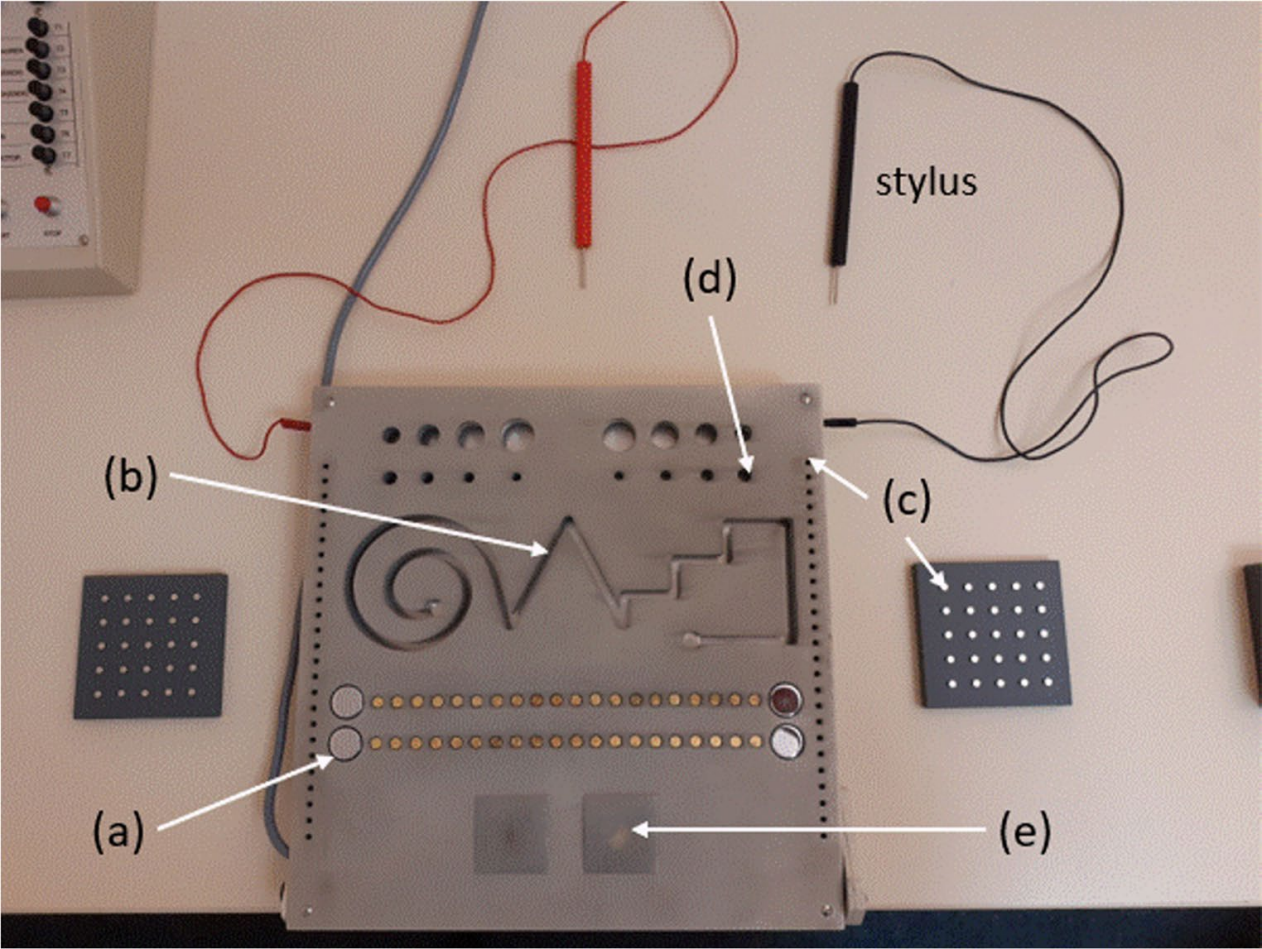
Top view on the working plate of the MLS. The figure shows the parts of the working plate that are required for different subtests of the MLS. For details see main text

We used the *MLS* (German acronym for „Motorische Leistungsserie“)^3^ for assessing different visuomotor skills in five different tasks. The hardware of the MLS consists of a metal box measuring 30 cm wide, 30 cm long, and 1.5 cm high (see Fig. 1). The central part of the MLS consists of the working plate on top of the box. The working plate contains several holes, contact plates, and a long, winding slit. In addition, two styluses are connected to the MLS, one to the upper left corner, and one to the upper right corner of the box. Each stylus carries current, and the MLS registers a contact between the stylus and a particular part of the plate. Moreover, there are two small blocks carrying 25 pegs each. One block carries 25 short pegs; the other block carries 25 long pegs. On each block, the pegs are presented in a 5 × 5 arrangement, each peg sticking in a hole.

The *d2-R* test (Brickenkamp et al., 2010) consists of a single sheet of paper. The test sheet contains 14 rows of 57 stimuli each. There are three possible targets (letter d with two dashes), and 13 possible distractors (letter d with one, three, or four dashes; letter p with two dashes). In each stimulus row, targets and distractors are presented in random order, with the constraint that rows 4–14 are actually repetitions of rows 1–3. The test sheet contains 308 targets, and 376 distractors, but row 1 is omitted from the analysis.

The *FAIR-2* test (Moosbrugger & Oehlschlägel, 2011) is delivered as an eight-page booklet. Examples of stimuli and instructions are presented on pages 2 and 3. The actual test sheets are presented on page 5 and page 7. Each test sheet contains 16 rows of 20 stimuli each. There are two possible targets, and two possible distractor stimuli. Each stimulus consists of an outer circle that contains either a smaller circle or a square, which in turn contains either two or three dots. The diameter of the outer circle of each stimulus is approximately 7 mm; the diameter of the inner circle or square is approximately 5 mm. In test version A, a circle with three dots and a square with two dots are targets, whereas a circle with two dots and a square with three dots are distractors. In test version B, a circle with two dots and a square with three dots are targets, whereas a circle with three dots and a square with two dots are distractors. Together, the two test pages contain 320 targets, and 320 distractors.

### Procedure

Each participant was tested individually in a single session. At the beginning of the session, participants received written information about the experiment (and a brief description of the tasks), and gave their informed consent to participate. Each participant then completed seven tasks (d2-R, FAIR-2, and five subtests of the MLS) in a predetermined order. In particular, there were 12 different sequences of tasks, and each sequence was assigned to 10 participants. The sustained-attention tests (d2-R, FAIR-2) were administered either both at the beginning, or both administered at the end, or one test at the beginning, and the other one at the end. Each of these possibilities was combined with two possible sequences of MLS subtasks (steadiness, line-tracking, pegboard task, tapping, aiming vs. aiming, tapping, pegboard task, line-tracking, steadiness).

For the *d2-R test*, participants were equipped with a pen, and received a sheet of paper containing written instructions, and two short stimulus rows for practice. Instructions informed participants that they were supposed to search each row from left to right for targets among irrelevant distractors, and to mark each target with a single short stroke. Moreover, participants were informed that they would be given 15 s^4^ per row after which the experimenter would say „Stop. Next row“. On this command, the participant was expected to stop working on the current row, and to immediately start searching (from left to right) on the next row. When the participant had finished practice, he or she was given the test sheet, and the experimenter gave the command to start working on the first row. After 15 s, the experimenter said „Stop. Next row,“ and this procedure was repeated until the participant had completed the entire test.

For the FAIR-2 test, participants were again equipped with a pen, and received the test booklet. Participants first completed some demographic information (age, gender, and highest degree) on the front page of the booklet. Next, participants turned to pages 2–3, which contained instructions, and a single row of stimuli for practicing the task. Instructions informed participants that they were supposed to search each row from left to right for targets among irrelevant distractors. Moreover, participants were told that during search they were supposed to draw a continuous line directly below the currently searched stimulus row. The line should pass below distractors, whereas a target should be marked by drawing an upward spike into the target. Moreover, participants were told that they would be given 3 minutes each for searching two pages with 16 rows of stimuli. After three minutes, the experimenter would say „Stop. Turn the page“. On this command, the participant was expected to stop working on the first page, turn the page, and to immediately start searching (at the upper left corner) the first row of the second page. When the participant had read instructions, and finished practice, the experimenter said „Turn the page. Go!”, and the participant started searching through the first page. The test then proceeded as instructed, and described above.

For the MLS *aiming* task, participants used the stylus with their dominant hand. The targets for the aiming task consisted of twenty brass disks, arranged in a horizontal row on the working plate (cf. (a) in Fig. 1). The diameter of a target disk was 5 mm, the horizontal distance between two adjacent disks was 4 mm. To the left and to the right of the target plates was a start disk (diameter 10 mm). The participants’ task was to put the stylus on the start disk (on the side of the dominant hand), and then to consecutively touch each target disk as quickly as possible, without touching the working plate around the target disks. The MLS registered (a) the time required for touching all target disks in the correct order, and (b) the number of errors (i.e. how often the stylus touched the working plate surrounding the target disks).

For the *line*-*tracking* task, participants again used the stylus with their dominant hand. The task required participants to put the stylus into the beginning of a long, winding slit (cf. (b) in Fig. 1), and then to move the stylus along the slit as quickly as possible without touching the walls of the slit, until the end of the slit was reached. The MLS registered (a) the time required for moving the stylus through the slit, and (b) the number of errors (i.e. how often the stylus touched the walls of the slit).

For the *pegboard* task, we used the set of 25 small pegs. Therefore, the block carrying the 25 small pegs was positioned 10 cm to the right or left (corresponding to dominant hand of the participants) of the working plate. For right-handers, the 25 target holes for the pegs were arranged vertically along the right edge of the working plate (cf. (c) in Fig. 1). For left-handers, the target holes were arranged vertically along the left edge of the working plate. The participants’ task was to take each peg from the block and to put it into a target hole on the working plate as quickly as possible. The pegs had to be filled into the holes from top to bottom. The MLS registered the time between putting a peg into the first hole (on the top) and putting a peg into the last hole (at the bottom).

For the *steadiness* task, participants used the stylus with their dominant hand. The target for the steadiness task was one of the holes on the upper edge of the working plate. There were eight target holes with different diameters (between 4.8 and 20 mm) for the right hand in the upper right corner of the working plate, and eight target holes with corresponding diameters for the left hand in the upper left corner of the working plate. We used a target hole with a diameter of 8.5 mm (cf. (d) in Fig. 1), as recommended in the manual. The participants’ task was to put the stylus into the target hole, and to hold the stylus for 32 s in a constant position without touching the inner walls of the hole. The MLS registered the number and duration of errors (i.e., touches of the inner wall of the target hole).

For the *tapping* task, participants again used the stylus with their dominant hand. The target for the tapping task was one of two square plates (40 × 40 mm) on the lower edge of the working plate. Righthanders used the right target plate (cf. (e) on Fig. 1); lefthanders used the left target plate. The participants’ task was to tap as often as possible (with the stylus) on the target plate in a period of 32 s. The MLS counted the number of taps on the target plate that occurred within 32 s.

Previous investigations, as reported in Neuwirth and Benesch (2012), have shown that performance in the five subtests of the MLS can correlate to different degrees, depending on the dependent variable (DV), and characteristics of the sample (such as age, or health condition). For the ten correlations between the five MLS subtests that are analyzed in our study, Neuwirth and Benesch (2012) reported correlations ranging between − 0.03 (line tracking × tapping) and − 0.38 (pegboard × tapping). We will report the correlations from the previous studies together with the correlations obtained in our study for comparison.

One might wonder why participants had the opportunity to practice both the d2-R and the FAIR-2, but did not practice the subtests of the MLS. This difference resulted from following the prescribed procedures for conducting these tests. In our view, the MLS subtests do not require practice because the tasks are quite simple, and easily explained. Hence, we do not believe that a short practice before each subtest of the MLS would have altered the results.

The experimenter stayed in the laboratory throughout the experiment. The experimenter read the instructions for each task of the MLS to the participant, and recorded the results of each task from the MLS display on a form that had been prepared for each participant. After the final test, participants were thanked and received payment or a certificate of participation. A complete session lasted approximately 45 min. Participants had the opportunity to take short breaks between two tests, in order to prevent fatigue.

### Design and data analysis

We had planned to analyze our dataset in three steps. First, we planned to compute descriptive statistics for all performance measures in each task. In the d2-R task, we planned to determine KL (i.e. hit rate) as a measure of speed, and the percentage of errors as a measure of accuracy. KL is simply the sum of correctly marked targets. In the FAIR-2 task, we planned to determine L as a measure of speed, and Q as a measure of accuracy. According to Moosbrugger and Oehlschlägel (2011), L is the sum of correctly inspected stimuli (i.e., hits + correct rejections) minus two times the sum of errors (false alarms + misses). Q is the proportion of correctly processed items (hits + correct rejections) relative to all inspected items. For the aiming task and the line-tracking task, we planned to measure the time (in sec.) required for completing the task, and the number of errors. In the pegboard task, we planned to measure the time for completing the task (in sec.) only. In the steadiness task, we planned to measure only the number of errors. Finally, in the tapping task, we counted the number of taps in a given period of time (i.e. 32 s.).

As a second analysis, we planned to compute and analyze correlations between performance measures observed in different tests. The correlational analysis was designed to answer three questions: First, are there substantial correlations between corresponding performance measures (i.e., KL and L; error percentage and Q) in the d2-R and the FAIR-2? Second, are there significant correlations between performance measures in different subtests of the MLS? Third, and most interestingly, are there significant correlations between performance measures in MLS tasks and performance measures in sustained-attention tests (d2-R, FAIR-2)?

As a third analysis, we planned to perform a multiple regression analysis with performance measures in the MLS subtests as predictor variables, and the speed measure from a sustained-attention test (d2-R: KL, FAIR-2: L) as a criterion variable. The regression analysis was designed to answer three questions. First, does performance in five subtests of the MLS (i.e., measures of motor skills) predict a significant part of the variance in performance in two different sustained-attention tests? Second, does performance in the MLS (i.e., measures of motor skills) predict more variance in performance in the FAIR-2 than in the d2-R? Third, which particular subtests of the MLS make a significant contribution to predicting performance in the d2-R and the FAIR-2?

## Results

### General performance

As a first step, we computed descriptive statistics for all performance measures that had been collected in our study. In the d2-R, participants achieved, on average, 129 hits, CI_95_ = [124.01; 133.29], while the error percentage amounted to 13.04%, CI_95_ = [10.94; 15.13]. In the FAIR-2 test, participants achieved an average L score of 413, CI_95_ = [398.09; 427.83], with an average accuracy score of 0.93, CI_95_ = [0.92; 0.94]. Descriptive statistics for performance on the d2-R and the FAIR-2 are presented in Table 1.

**Table 1:** Means of performance scores obtained in the tests d2-R and FAIR-2. Standard deviations are given in parentheses

d2 (hits)	d2 (error %)	FAIR-2 (L)	FAIR-2 (Q)
129.10 (23.18)	13.04 (11.58)	412.96 (82.29)	0.93 (0.05)

Note. The maximal number of hits in the d2-R is 286. The maximal number of L in the FAIR-2 is 640

Descriptive measures of performance in different tasks of the MLS (excluding tapping) are presented in Table 2. In the aiming task, participants required, on average, 8.63 s, CI_95_ = [8.18; 9.08], to touch all targets, while errors were rare, *M* = 0.52, CI_95_ = [0.33; 0.71]. There was a negative correlation between time and errors in the aiming task (*r* = −.309, *p* <.001), indicating a speed-accuracy tradeoff. In the pegboard task, participants required, on average, 48.3 s, CI_95_ = [46.24; 50.28], to put all pegs into their holes. In the steadiness task, participants made, on average, 9.6 errors, CI_95_ = [7.87; 11.29]. In the line-tracking task, participants required, on average, 43.13 s, CI_95_ = [39.82; 46.43], to complete the track, while making, on average, 20 errors, CI_95_ = [18.17; 21.88]. Time and errors were not significantly correlated in the line-tracking task (*r* =.097, *p* =.292). Finally, in the tapping task, participants made, on average, 212 taps, CI_95_ = [207.66; 216.06].

**Table 2:** Means of performance scores for the present sample, and a reference sample, as obtained in four subtests of the MLS. Standard deviations are given in parentheses. Time is given in seconds (sec)

	Present Sample			Reference sample	
	Time (sec)	Errors		Time (sec)	Errors
Aiming	8.63 (02.50)	0.52 (01.05)		8.74 (2.18)	1.00 (1.97)
Pegboard	48.26 (11.18)			39.20 (5.09)	
Steadiness		9.58 (09.46)			11.39 (18.37)
Line-tracking	43.13 (18.28)	20.03 (10.27)		23.23 (10.36)	27.36 (11.99)

Note. The data for the reference sample (*N* = 420, Age = 18–60) are taken from Neuwirth and Benesch (2012; p.20)

### Correlational analysis

In a second step, we computed Pearson correlation coefficients between the major performance scores in the five subtests of the MLS, and the four performance measures obtained in the d2-R and the FAIR-2. For the two MLS tasks (aiming and line-tracking) that provide both time (speed) and error measures, we decided to use only the speed measure for our analysis. The resulting correlation matrix can be found in Table 4 in the appendix. We briefly summarize here the significant correlations observed (a) between performance measures in the d2-R and the FAIR-2, (b) between performance measures in different subtests of the MLS, and (c) between performance in subtests of the MLS and performance measures in the d2-R and the FAIR-2, respectively.

In our correlational analysis, many correlation coefficients are tested against zero by one-sample *t* tests. Hence, one may ask if these multiple tests are compromised by alpha-error inflation, and if thus the significance criterion α should be reduced accordingly. In fact, however, the problem of alpha-error inflation is restricted to situations where a group (or family) of tests are used in concert to test a single, overarching null hypothesis, and each single test could falsify this omnibus null hypothesis (cf. García-Pérez, 2023; Rubin, 2021). This precondition for an alpha-error correction is not met here. Rather, testing correlation coefficients against zero in our study is intended to determine which subtest of the MLS covaries with performance in the d2-R and FAIR-2, respectively, and which does not. From this perspective, the individual tests are logically independent and do not require a correction of α (García-Pérez, 2023; Rubin, 2021).

There were significant within-test correlations between speed and accuracy measures. For the d2-R, hits and error percentages were negatively correlated (*r* = −.470, *p* <.001, CI_95_ = [−0.611; − 0.329])^5^. That is, as the number of hits increased, the number of misses and false alarms decreased. Similarly, in FAIR-2, L and Q (i.e. accuracy) were positively correlated (*r* =.352, *p* <.001, CI_95_ = [0.193; 0.511]). There were also some significant between-test correlations. In particular, the speed measures of both tasks (i.e. hits and L) were positively correlated (*r* =.534, *p* <.001, CI_95_ = [0.405; 0.664]), and the accuracy measures of both tasks (i.e., error percentage, Q) were negatively correlated (*r* = −.322, *p* <.001 CI_95_ = [−0.484; − 0.159]).

There were three moderate correlations between performance in different subtests of the MLS. Positive correlations occurred between aiming time and pegboard time (*r* =.183, *p* =.045, CI_95_ = [0.008; 0.358]), and between pegboard time and steadiness errors (*r* =.195, *p* =.032, CI_95_ = [0.021; 0.369]). For comparison, the corresponding values reported by Neuwirth and Benesch (2012) were 0.24 and 0.18, respectively. A negative correlation occurred between aiming time and the number of taps in the tapping task (*r* = −.185, *p* =.043, CI_95_ = [0.010; 0.036]). The corresponding value reported by Neuwirth and Benesch (2012) was − 0.18.

Finally, there were some significant correlations between performance measures in the MLS subtests, and performance in the d2-R and FAIR-2, respectively. Concerning the d2-R, there was a positive correlation between the number of hits and the number of taps in the tapping task (*r* =.201, *p* =.028, CI_95_ = [0.027; 0.375]), and a negative correlation between error percentage and the time required for completing the line-tracking task (*r* = −.290, *p* =.001, CI_95_ = [−0.456; − 0.124]). Concerning the FAIR-2, the speed (or quantity) measure L was positively correlated with the number of taps in the tapping task (*r* =.221, *p* =.015, CI_95_ = [0.049; 0.393]), but negatively correlated with the time required for the pegboard task (*r* = −.233, *p* =.011, CI_95_ = [−0.404; − 0.062]). The accuracy measure Q was positively correlated with the time taken to complete the line-tracking task (*r* =.186, *p* =.042, CI_95_ = [0.011; 0.361]), but negatively correlated with errors in the steadiness task (*r* = −.239, *p* =.009, CI_95_ = [−0.409; − 0.068]), and with the time needed for the pegboard task (*r* = −.181, *p* =.048, CI_95_ = [−0.356; − 0.006]).

### Regression analysis

In a third step, we performed multiple linear regression analyses with performance in five subtests of the MLS (aiming time, pegboard time, line-tracking time, steadiness errors, and number of taps in the tapping task) as predictor variables and a performance measure from the d2-R and the FAIR-2, respectively, as the criterion variable. Note that we had pre-registered the multiple regression analyses for the speed (or quantity) measures from the d2-R (KL/hits) and the FAIR-2 (L), but not for the error or accuracy measures. Nevertheless, we will report the results for all four regression analyses, although the latter two were not pre-registered, and are therefore considered exploratory analyses. The order of entering the predictor variables into the analysis was determined by the size of the correlation with the criterion variable, with the predictor having the highest correlation being entered first, and so on.

In the context of each regression analysis, many individual t tests are conducted in order to test regression coefficients against zero. Here again, one may ask if these multiple tests are compromised by alpha-error inflation. However, the answer to this question is again negative. The reason (again) is that the tests of the regression coefficients do *not* serve the goal of testing one overarching null hypothesis (i.e. the hypothesis whether the whole model explains a significant amount of variance of the criterion variable), which would justify a family-wise correction of alpha levels. In fact, this hypothesis is already tested in a single F test. In contrast, testing the regression coefficients against zero serves to answer the question of whether a particular predictor variable explains a significant amount of variance of the criterion variable or not. Hence, again, the individual tests are logically independent and do not require a correction of α (García-Pérez, 2023; Rubin, 2021).

We checked the main assumptions for all four regression analyses. First, these checks showed that multicollinearity was not a concern (0.918 ≤ Tolerance ≤ 0.974; 1.027 ≤ VIF ≤ 1.090). Furthermore, all data sets also met the assumption of independent errors (1.716 ≤ Durbin-Watson ≤ 2.222). An inspection of the scatterplots of standardized residuals suggested linearity and homoscedasticity (i.e., balanced distribution of residuals depending on predicted values) for most of the criterion variables, except for the d2 error percentage (relevant scatterplots for all analyses can be found in the Supplemental Materials). The Shapiro-Wilk test showed that the distribution of residuals did not deviate from normality for FAIR L, *W* = 0.987, *p* =.332. In contrast, the distribution of residuals deviated significantly from normality for d2 hits, *W* = 0.975, *p* =.027, d2 error percentages, *W* = 0.890, *p* <.001, and FAIR Q, *W* = 0.821, *p* <.001. Screening for influential data points and outliers revealed that Cook’s distance was inconspicuous for all analyses (cf. Table 3). Therefore, we decided to run the regression analyses on the full data set.

**Table 3:** Values of Cook’s distance for the multiple regression analyses reported here

Criterion variable	Mean	SD	Maximum
d2 – Hits	0.012	0.031	0.253
d2 – Error Percentage	0.009	0.019	0.127
d2 – BIS	0.011	0.026	0.185
FAIR – L	0.009	0.016	0.110
FAIR – Q	0.011	0.039	0.348
FAIR – BIS	0.010	0.024	0.178

The first regression analysis tested whether performance in the five subtests of the MLS significantly predicted the number of hits in the d2-R. The obtained regression parameters are shown in Table 5 in the appendix. The overall regression model was not statistically significant, *R*² = 0.056, CI_95_ = [−0.019; 0.132]^6^, *F*(5, 114) = 1.356, *p* =.246.

The second regression analysis tested whether performance in the five subtests of the MLS significantly predicted the speed measure L of the FAIR-2. The obtained regression parameters are shown in Table 6 in the appendix. The overall regression model was statistically significant, *R*² = 0.116, CI_95_ = [0.014; 0.218], *F*(5, 114) = 3.004, *p* =.014. The predictor variables pegboard time, *t*(119) = −2.145, *p* =.034, and number of taps, *t*(119) = 2.191, *p* =.030, significantly contributed to predicting L, whereas the remaining variables did not, all *t*s(119) < 1.2, all *p*s > 0.20.

The third regression analysis tested whether performance in the five subtests of the MLS significantly predicted the error percentage PE in the d2-R. The obtained regression parameters are shown in Table 7 in the appendix. The overall regression model was statistically significant, *R*² = 0.097, CI_95_ = [0.002; 0.193], *F*(5, 114) = 2.439, *p* =.039. The predictor line-tracking time, *t*(119) = −3.061, *p* =.003 significantly contributed to predicting PE, whereas the remaining variables did not, all *t*s(119) < 1.2, all *p*s > 0.200.

Finally, the fourth regression analysis tested whether performance in the five subtests of the MLS significantly predicted the accuracy measure Q obtained with the FAIR-2. The corresponding regression parameters are shown in Table 8 in the appendix. The overall regression model was statistically significant, *R*² = 0.107, CI_95_ = [0.008; 0.206], *F*(5, 114) = 2.736, *p* =.023. The predictor variable steadiness errors, *t*(119) = −2.093, *p* =.039 significantly contributed to predicting Q, whereas the remaining variables did not, all *t*s(119) < 2.0, all *p*s > 0.050.

In a final analysis, we compared the size of the variance (R²) that performance in the MLS explained in d2-R performance with the size of the variance that MLS performance explained in FAIR-2 performance. R² can be conceptualized as the correlation between a set of predictors and a criterion variable (e.g., Tabachnik & Fidell, 2013). Hence, we used a test for comparing correlation coefficients from dependent samples, suggested by Meng et al. (1992)^7^ for that purpose. This test showed that the variance explained by MLS performance in the speed measures of d2-R (*R*² = 0.056) and FAIR-2 (*R*² = 0.116) did not differ, z = − 0.546, *p* =.585 (two-tailed). Similarly, the variance explained by MLS performance in the accuracy measures of d2-R (*R*² = 0.097) and FAIR-2 (*R*² = 107) did not differ, z = −0.083, *p* =.935 (two-tailed). The results of the Meng test are corroborated by overlapping confidence intervals of the to-be-compared measures.

### Exploratory analysis: regression analysis with BIS as dependent variable

Because the speed and accuracy measures of both tests were significantly correlated, we combined the measures for each test in a combined measure, the Balanced integration Score (BIS; Liesefeld et al., 2015). The BIS removes the impact of speed-accuracy tradeoffs on a performance measure, and recent studies showed that it does so more efficiently than alternative measures, such as the inverse-efficiency score (Liesefeld & Janczyk, 2019). Essentially, the BIS is computed by subtracting the z-transformed accuracy measure (i.e., proportion correct, or *PC*) from the z-transformed speed measure (e.g., RT, sum of hits). For the FAIR-2 test, a speed measure (L) and a PC measure (Q) are already available from the standard analysis of the test. In contrast, for the d2-R test, the speed measure (hits) is available, but the PC measure had to be computed from the data. For computing the PC measure, we first determined the sum of inspected items (i.e., the number of items to the left of the right-most marked target), and the sum of errors for each participant, and computed PC according to the formula $\:\frac{sum\:of\:inspected\:items-sum\:of\:errors}{sum\:of\:inspected\:items}$. Subsequently, we conducted multiple regression analyses with the five performance measures from the MLS as predictor variables, and the BIS measure for each test as the criterion variable.

An initial multiple linear regression analysis tested whether performance in the five subtests of the MLS significantly predicted the BIS measure for the d2-R. The overall regression model was not statistically significant, R² = 0.055, CI_95_ = [−0.020; 0.130], *F*(5, 114) = 1.322, *p* =.260. Tests of collinearity showed that multicollinearity was not a concern (all Tolerances between 0.91 and 0.98, all *VIF* values between 1.0 and 1.1). In addition, the data met the assumption of independent errors (Durbin-Watson value = 1.861). Inspection of scatter plots suggested linearity and homoscedasticity of residuals (scatter plots can be found in the Supplemental Materials). However, the Shapiro-Wilk test showed that the distribution of residuals deviated from normality, *W* = 0.923, *p* <.001. Detailed results of this regression analysis are shown in Table 9 in the appendix.

A second multiple linear regression analysis was performed to determine whether performance in the five subtests of the MLS significantly predicted the BIS measure for the FAIR-2. The overall regression model was statistically significant *R*² = 0.132, CI_95_ = [0.025; 0.239], *F*(5, 114) = 3.481, *p* =.006. The predictor variables line-tracking time, *b* = −0.014, *SE* = 0.05, β = −0.241; *t*(119) = −2.728, *p* =.007, and number of taps, *b* = 0.010, *SE* = 0.004, β = 0.211; *t*(119) = 2.336, *p* =.021, significantly contributed to predicting BIS_FAIR_, whereas the remaining variables did not, all *t*s(119) < 1.4, all *p*s > 0.150. Assumption checks indicated that the assumptions were met (scatter plots can be found in the Supplemental Materials). Tests of collinearity indicated that multicollinearity was not a problem (all tolerances between 0.91 and 0.98, all *VIF* values between 1.0 and 1.1). Moreover, the data met the assumption of independent errors (Durban-Watson value = 2.129). Inspection of scatter plots suggested linearity and homoscedasticity of residuals. Finally, the Shapiro-Wilk test showed that the distribution of residuals did not significantly deviate from normality, *W* = 0.985, *p* =.195. Detailed results of this regression analysis are shown in Table 10 in the appendix.

Finally, we compared the size of the variance (R²) that performance in the MLS explained in the two BIS measures representing d2-R and FAIR-2 performance. The Meng test showed that the variance explained by MLS performance in the two BIS measures did not differ, z = − 0.686, *p* =.493 (two-tailed). Again, the results of the Meng test are corroborated by overlapping confidence intervals of the to-be-compared measures.

## Discussion

A first aim of the present study was to investigate whether motor skills have a significant impact on performance in the d2-R and the FAIR-2, two pen-and-paper tests of selective attention. In addition, we wanted to gain a better understanding of the particular motor skills that are required for the d2-R and the FAIR-2. Previous studies have reported correlations between performance in simple motor tasks and performance on the d2. These studies reported correlations between reaction time to simple visual stimuli and KL (e.g., Schwalbach in prep., as cited in Brickenkamp et al., 2010). Other studies have also found correlations between the number of marked circles on a sheet of paper and KL (e.g., Enders, 2004). Neither simple reaction time nor the number of marked circles reveals very specific insights into the motor demands that are required by working on the d2-R or the FAIR-2. In order to gain more specific insights, we used the MLS which consists of five motor tasks with different demands.

Target marking in the d2-R requires discrete, goal-directed movements to target stimuli. Hence, we expected that performance in the MLS subtests aiming and tapping, which require discrete, goal-directed movements, would correlate more strongly with performance in the d2-R than performance in other subtests. These predictions were partly confirmed by observing a positive correlation between tapping and KL, while there was no correlation between aiming and KL. Performance in the remaining subtests of the MLS also failed to correlate with KL, and therefore a multiple regression model, with all subtests of the MLS as predictor variables and KL as the criterion variable, was not significant (*R*² = 0.056). The only significant correlation between the MLS and the error percentage in the d2-R was a negative correlation between line-tracking time and PE. This correlation was high enough, however, that a multiple regression model, with all subtests of the MLS as predictor variables and PE as the criterion variable, was significant (*R*² = 0.097). In summary, we observed only weak correlations between motor skills and performance in the d2-R.

This is the first study to investigate the impact of motor skills on performance in the FAIR-2 test. Due to the requirement of continuous marking in the FAIR-2, we expected that performance in the MLS subtest line-tracking would have the highest correlation among the MLS subtests with performance in the FAIR-2. This prediction was partly confirmed by observing a correlation between line-tracking time and Q (i.e. percentage correct), whereas the correlation between line-tracking time and L (i.e. hits) was not significant. However, L correlated significantly with pegboard time and tapping hits. Moreover, Q was also correlated with steadiness errors and pegboard time. A multiple regression model, with all subtests of the MLS as predictor variables and L as the criterion variable, was significant (*R*² = 0.116). In addition, a multiple regression model with Q as the criterion variable was also significant (*R*² = 0.107).

### Qualitative differences in the effects of motor skills on attention-test performance

Comparing the correlations between performance in the MLS subtests and performance measures in the d2-R and the FAIR-2, respectively, allowed for analyzing the motor demands of the two tests in qualitative terms. We observed both commonalities and differences in motor demands between the two tests. The commonalities were that tapping hits correlated with the speed measures (i.e., hit rates KL and L) in both tests, and line-tracking time correlated with accuracy measures (i.e., PE and Q) in both tests. The tapping task is a rather simple, repetitive task for which we had not expected a big effect on performance in either test. However contrary to our expectation, the tapping task seems to capture a facet of behavior that is also required in the two tests. This facet may simply involve making rapid, repetitive movements with a pen. In contrast, the line-tracking task requires to perform a continuous movement with high accuracy. At first sight, the requirements of the line-tracking task seem to resemble the requirements of the FAIR-2 more strongly than those of the d2-R. At closer inspection, however, the d2-R also requires continuous movements with high precision. In the d2-R, participants have to continuously search a row of stimuli, and most of them will move their pen along the currently inspected row to have it close to the next target if one is detected.

Our study also revealed some differences between the motor demands of the two tests. A first difference concerned the variable *pegboard* time which correlated with both speed and accuracy in the FAIR-2, while not correlating with performance in the d2-R. A second difference involved the variable *steadiness* errors that correlated with accuracy measure Q in the FAIR-2, but did not correlate with performance in the d2-R. The *pegboard* task imposes high demands on eye-hand coordination when executing goal-directed movements. Apparently, these demands are also required in the FAIR-2 when participants have to make precise, goal-directed movements for drawing spikes towards the target stimuli. In contrast, the *steadiness* task requires continuous control of hand posture, and the continuous control of hand posture may also be required when drawing a smooth and continuous line in the FAIR-2.

### Quantitative differences in the effects of motor skills on attention-test performance

The results of the multiple regression analyses allowed for a quantitative comparison of motor demands on performance in the two tests of sustained attention. These analyses showed that the five subtests of the MLS explained more variance of performance parameters for the FAIR-2 (*R*² = 0.116, for L, *R*² = 0.107 for Q) than for the d2-R (*R*² = 0.056 for KL, *R*² = 0.097 for PE). Similarly, multiple regression analyses with the combined speed-accuracy measure BIS (Liesefeld et al., 2015) suggested that motor skills, as measured by the MLS, explain twice as much variance of performance in the FAIR-2 (*R*² = 0.13) than in the d2-R (*R*² = 0.06). However, the numerical differences in explained variance were not statistically significant. Therefore, we conclude that the complete set of fine motor skills, which are measured by the MLS, affect performance in the FAIR-2 and the d2-R to a similar, moderate degree, although more fine-grained analyses revealed some qualitative differences in the motor requirements between the tests. In other words, our results indicate that the sum of motor demands in the d2-R and the FAIR-2 are comparable, although there are some differences in specific motor requirements.

### On the validity of the d2-R and the FAIR-2

The present study can also be seen as a study on the convergent and discriminant validity (Campbell & Fiske, 1959) of the d2-R and the FAIR-2. Both the d2-R and the FAIR-2 are intended to measure selective visual attention, and both tests use a visual conjunction-search task for assessing this ability. Hence, performance of the same persons in the two tests should be highly correlated, reflecting convergent validity. Previous studies have typically reported correlations of intermediate size (i.e. *r* ≈.50) between speed measures in the d2-R (GZ-F, KL) and the speed measure L in the FAIR/FAIR-2 (e.g., Brickenkamp et al., 2010; Hallwachs, 1994; Moosbrugger & Oehlschlägel, 2011). We observed similar correlations between the d2-R and the FAIR-2. In particular, we observed a correlation of *r* =.53 between KL and L, and therefore replicate the previous studies. In addition, for accuracy, we observed a (negative) correlation of *r* = −.32 between PE (d2-R) and Q (FAIR-2), but we could not find correlations between accuracy measures from the two tests in the literature for comparison. Compared with the correlations observed between the d2-R and the FAIR-2, the correlations between these two tests and the subtests of the MLS are much smaller. Concerning the d2-R, correlations between the speed measure KL and subtests of the MLS varied between *r* = −.073 and *r* =.201, and correlations between the accuracy measure PE and subtests of the MLS varied between *r* = −.290 and *r* =.057. Concerning the FAIR-2, correlations between the speed measure L and subtests of the MLS varied between *r* = −.233 and *r* =.221, and correlations between the accuracy measure Q and subtests of the MLS varied between *r* = −.239 and *r* =.186.

We used the Meng test (Meng et al., 1992) to examine convergent and discriminant validity in more detail. Specifically, we compared the correlation between the speed measures from the d2-R (KL) and the FAIR-2 (L) with the correlations between these speed measures and the MLS. In terms of convergent and discriminant validity, we expected that the correlation between KL and L should be significantly higher than the highest correlations between these two measures and the MLS. The analysis revealed that the correlation between KL and L (*r* =.534) was higher than (a) the highest correlation between KL and the MLS, z = 3.233, *p* =.001, and (b) the highest correlation between L and the MLS, z = 3.021, *p* =.003. Next, we compared the correlation between the accuracy measures from the d2-R (PE) and the FAIR-2 (Q) to the correlations between these measures and the MLS. This analysis revealed that the correlation between PE and Q (*r* = −.322) differed neither from the correlation between PE and the MLS, z = −0.231, *p* =.771, nor from the correlation between Q and the MLS, z = −0.695, *p* =.487. We therefore conclude that the pattern of correlations between the speed measures (KL, L) and the MLS provides convergent validity for the d2-R and the FAIR-2, and discriminant validity for each of these tests and the MLS. This conclusion, however, does not hold for the accuracy measures (PE, Q).

### Power considerations

Before conducting our study, we performed a power analysis for determining our sample size where we focused on the regression analyses, and therefore we used R² as the DV for our power analysis. Yet, since we had planned, and performed, several sets of analyses, we could have made additional power analyses before conducting our study. For example, we could have also made power analyses for our correlational analyses, and for individual regression coefficients. For the sake of completeness, we performed a post-hoc power analysis to determine the sample size for correlations, and this analysis revealed a sample size of *N* = 115, which is very close to our sample size, when high power (1-β = 0.95) and an intermediate effect size (*r* =.30; Cohen, 1988) are assumed.

Since our statistical comparisons of R² values produced non-significant results, one might wonder if (a lack of) power might have affected these results. In fact, based on the results of simulation studies, Schönbrodt and Perugini (2013) suggested that—in typical scenarios—correlation coefficients would stabilize when sample size approaches 250, although the sample size is affected by the size of the correlation in the population. Therefore, we tested if the significance of the differences between the *R*² values observed in our study would have changed with a larger sample size (i.e. *N* = 250). This was not the case. However, this does not exclude the possibility that the results (i.e. correlation coefficients, R² values) might be different with a larger sample.

## Concluding remarks

According to Moosbrugger and Oehlschlägel (1996, 2011), the task of continuous marking in the FAIR-2 forces participants to follow instructions more closely as compared to the d2-R, and also provides the user of the FAIR-2 with better means to check whether participants followed the instructions. The greater control over the degree to which participants follow the instructions in the FAIR-2, as compared to the d2-R, might come with a cost however. In particular, continuous marking might increase the motor demands of the task when compared to discrete marking, and increased motor demands in turn could decrease the validity of the task for measuring attention. The results of our study, however, suggest that, although the motor demands of the two tests show some differences, the overall motor demands of the FAIR-2 are not significantly larger than those of the d2. Hence, when compared to the d2-R, the FAIR-2 provides more experimental control over the degree to which participants follow instructions while neither significantly increasing the motor demands nor decreasing the validity of the test.

## Appendix

Please see Tables 4, 5, 6, 7, 8, 9 and 10.

**Table 4:** Pearson correlations between five measures of motor skill, and results from the d2-R and the FAIR-2 (*N* = 120)

Measure	Coefficient	Aiming (t)	Steadiness (E)	Pegs (t)	Tapping	Line-tracking (t)	d2 (hits)	d2 (PE)	FAIR (L)
Aiming (t)	Pearson’s r	—							
	*p* value	—							
Steadiness (E)	Pearson’s r	−0.033	—						
	*p* value	0.719	—						
Pegs (t)	Pearson’s r	**0.183**	**0.195**	—					
	*p* value	**0.045**	**0.032**	—					
Tapping	Pearson’s r	**−0.185**	−0.160	−0.041	—				
	*p* value	**0.043**	0.081	0.657	—				
Line-tracking (t)	Pearson’s r	0.074	−0.113	0.056	0.067	—			
	*p* value	0.420	0.219	0.542	0.465	—			
d2 (hits)	Pearson’s r	−0.044	−0.068	−0.073	**0.201**	0.116	—		
	*p* value	0.636	0.462	0.426	**0.028**	0.208	—		
d2 (PE)	Pearson’s r	−0.119	0.057	−0.034	−0.054	**−0.290**	**−0.470**	—	
	*p* value	0.194	0.539	0.712	0.556	**0.001**	**< 0.001**	—	
FAIR (L)	Pearson’s r	−0.156	−0.095	**−0.233**	**0.221**	−0.103	**0.534**	−0.060	—
	*p* value	0.089	0.300	**0.011**	**0.015**	0.261	**< 0.001**	0.512	—
FAIR (Q)	Pearson’s r	0.020	**−0.239**	**−0.181**	0.011	**0.186**	0.173	**−0.322**	**0.352**
	*p* value	0.832	**0.009**	**0.048**	0.906	**0.042**	0.059	**< 0.001**	**< 0.001**

Note. E = errors; t = time; PE (percentage of errors); significant coefficients (i.e., *r* < 0) are printed in bold

**Table 5:** Regression coefficients obtained in multiple linear regression analyses with d2-R hits as the criterion variable. Significant coefficients are printed in bold

Predictor Variable	b	SE	CI_95_ lower	CI_95_ upper	t	*p*	β
Intercept	**91.011**	24.270	42.933	139.089	3.750	< 0.001	
Aiming Time	−0.042	0.879	−1.783	1.700	−0.047	0.962	− 0.004
Line-tracking Time	0.134	0.117	−0.098	0.366	1.147	0.254	0.106
Number of Taps	**0.188**	0.094	0.002	0.374	1.999	0.048	**0.188**
Pegboard Time	−0.142	0.197	−0.531	0.248	−0.720	0.473	−0.068
Steadiness Errors	−0.031	0.233	−0.492	0.430	−0.133	0.895	−0.013

Note. SE: Standard error (of the unstandardized coefficient b)

**Table 6:** Regression coefficients obtained in multiple linear regression analyses with L from the FAIR-2 as the criterion variable. Significant coefficients are printed in bold

Predictor Variable	b	SE	CI_95_ lower	CI_95_ upper	t	*p*	β
Intercept	**378.270**	83.373	213.108	543.432	4.537	< 0.001	
Aiming Time	−2.516	3.019	−8.498	3.465	−0.833	0.406	−0.076
Line-tracking Time	−0.470	0.402	−1.266	0.325	−1.172	0.244	−0.105
Number of Taps	**0.707**	0.323	0.068	1.347	2.191	0.030	**0.200**
Pegboard Time	**−1.448**	0.675	−2.786	−0.111	−2.145	0.034	**−0.197**
Steadiness Errors	−0.343	0.799	−1.927	1.240	−0.430	0.668	−0.039

Note. SE: Standard error (of the unstandardized coefficient b)

**Table 7:** Regression coefficients obtained in multiple linear regression analyses with error percentages from the d2-R test as the criterion variable. Significant coefficients are printed in bold

Predictor Variable	b	SE	CI_95_ lower	CI_95_ upper	t	*p*	β
Intercept	**30.567**	11.866	7.061	54.073	2.576	0.011	
Aiming Time	−0.499	0.430	−1.350	0.352	−1.161	0.248	−0.108
Line-tracking Time	**−0.175**	0.057	−0.288	−0.062	−3.061	0.003	**−0.276**
Number of Taps	−0.027	0.046	−0.118	0.046	−0.581	0.562	−0.054
Pegboard Time	−0.004	0.096	−0.194	0.187	−0.040	0.968	−0.004
Steadiness Errors	0.017	0.114	−0.208	0.243	0.151	0.880	0.014

Note. SE: Standard error (of the unstandardized coefficient b)

**Table 8:** Regression coefficients obtained in multiple linear regression analyses with Q from the FAIR as the criterion variable. Significant coefficients are printed in bold

Predictor Variable	b	SE	CI_95_ lower	CI_95_ upper	t	*p*	β
Intercept	**0.968**	0.054	0.861	1.075	17.942	< 0.001	
Aiming Time	< 0.001	0.002	−0.003	0.004	0.249	0.804	0.023
Line-tracking Time	0.001	0.000	−0.000	0.001	1.932	0.056	0.173
Number of Taps	−0.000	0.000	−0.000	0.000	−0.370	0.712	−0.034
Pegboard Time	−0.001	0.000	−0.002	0.000	−1.717	0.089	−0.158
Steadiness Errors	**−0.001**	0.001	−0.002	−0.000	−2.093	0.039	**−0.193**

Note. SE: Standard error (of the unstandardized coefficient b)

**Table 9:** Regression coefficients obtained in multiple linear regression analyses with the BIS measure for the d2-R test as the criterion variable. Significant coefficients are printed in bold

Predictor Variable	b	SE	CI_95_ lower	CI_95_ upper	t	*p*	β
Intercept	−0.245	1.103	−2.429	1.939	−0.223	0.824	
Aiming Time	−0.047	0.039	−0.127	0.032	−1.192	0.236	−0.113
Line-tracking Time	−0.006	0.005	−0.017	0.005	−1.118	0.266	−0.103
Number of Taps	0.006	0.004	−0.003	0.014	1.363	0.176	0.129
Pegboard Time	−0.007	0.009	−0.024	0.011	−0.724	0.470	−0.069
Steadiness Errors	−0.001	0.011	−0.022	0.019	−0.102	0.919	−0.009

Note. SE: Standard error (of the unstandardized coefficient b)

**Table 10:** Regression coefficients obtained in multiple linear regression analyses with the BIS measure for the FAIR test as the criterion variable. Significant coefficients are printed in bold

Predictor Variable	b	SE	CI_95_ lower	CI_95_ upper	t	*p*	β
Intercept	−1.045	1.055	−3.135	1.045	−0.991	0.324	
Aiming Time	−0.038	0.038	−0.113	0.038	−0.981	0.329	−0.089
Line-tracking Time	**−0.014**	0.005	−0.024	−0.004	−2.728	0.007	**−0.241**
Number of Taps	**0.009**	0.004	0.002	0.018	2.336	0.021	**0.211**
Pegboard Time	−0.004	0.009	−0.021	0.013	−0.515	0.608	−0.047
Steadiness Errors	0.014	0.010	−0.006	0.034	1.388	0.168	0.126

Note. SE: Standard error (of the unstandardized coefficient b)

## Supplementary Information

Below is the link to the electronic supplementary material.

Supplementary Material 1

## Data Availability

The present research did not produce digital raw data files. Raw data consisted of test sheets completed by participants, and in test protocols where experimenters noted measures from the MLS. These test sheets and protocols will not be published, but Excel sheets with individual tests scores can be obtained from the authors upon request.

## Footnotes

1 FAIR is an acronym for „Frankfurter Aufmerksamkeits Inventar“ („Frankfurt Attention Inventory“).

2 Too many line errors indicate that a participant did not follow instructions as required, and would render a test invalid.

3 The German name „Motorische Leistungsserie“ can be translated as „Motor Performance Series“.

4 We decided to provide 15 rather than 20 s per row to avoid ceiling effects.

5 For computing the confidence intervals of correlations coefficients, we used a procedure published by Bonett and Wright (2000). An online calculator can be found at https://www.psychometrica.de/korrelation.html#confidence.

6 For computing confidence intervals for coefficients of determination, we used a procedure proposed by Olkin and Finn (1995). An online calculator is provided by Soper (2025).

7 The Meng test compares the correlations of two different variables (r_13_, r_23_) with a third variable. Therefore, the test also considers the correlation r_12_ between the two criterion variables in our case. An online calculator for this test can be found in Hahn (2025).

## Declarations

### **Preregistration**

We preregistered this study at OSF on November 3, 2021 (10.17605/OSF.IO/6A4W3).
