# Supplementary material for: The impact of visuomotor skills on two pen-and-paper tests of sustained attention (d2-R, FAIR)
Source: Psychol Res. 2025 Nov 28;89(6):180. doi: 10.1007/s00426-025-02198-x (PMC12662847; doi:10.1007/s00426-025-02198-x)
Supplement: Supplementary file 1 — Supplementary Material 1 [file 426_2025_2198_MOESM1_ESM.pdf]

Supplemental Material

for article

The impact of visuomotor skills on two pen-and-paper tests of sustained attention (d2-R, FAIR)

Peter Wühr, Bianca Wühr, & Gerhard Rinkenauer

1. Multiple Regression with d2 hits as criterion variable

1.1 Normality

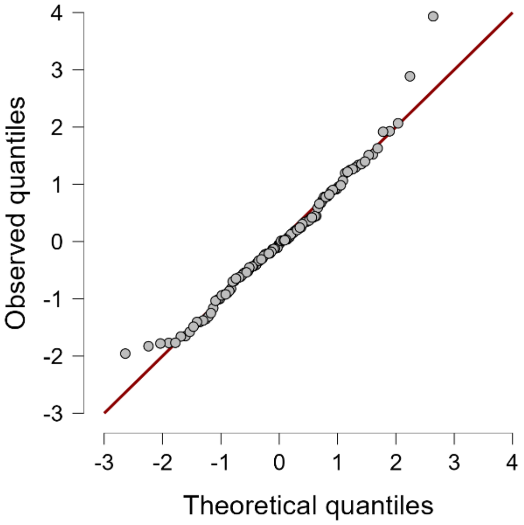

1.2 Homoscedasticity

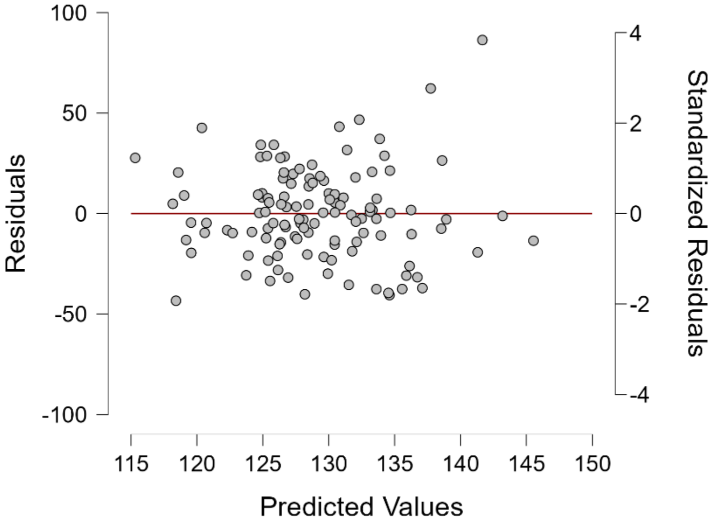

1.3 Linearity

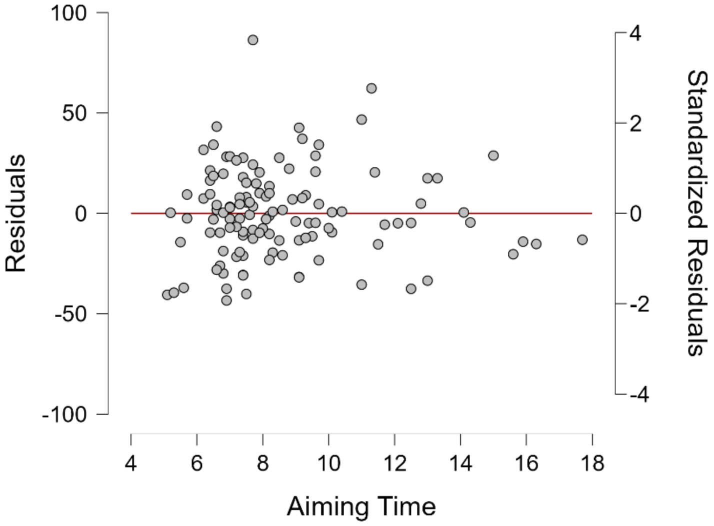

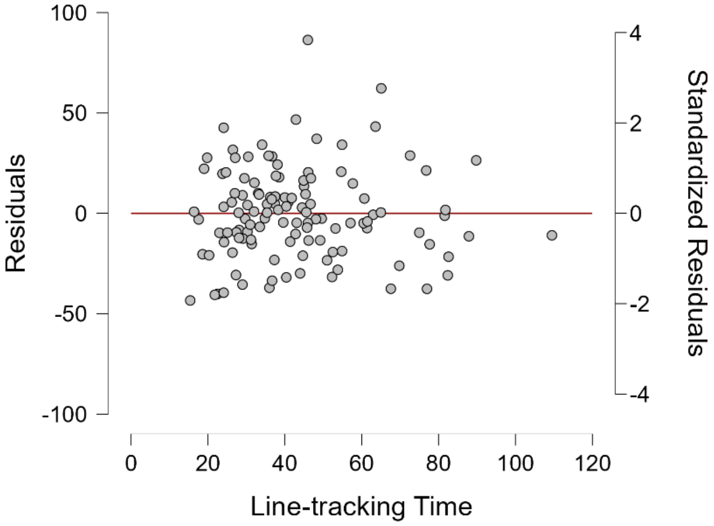

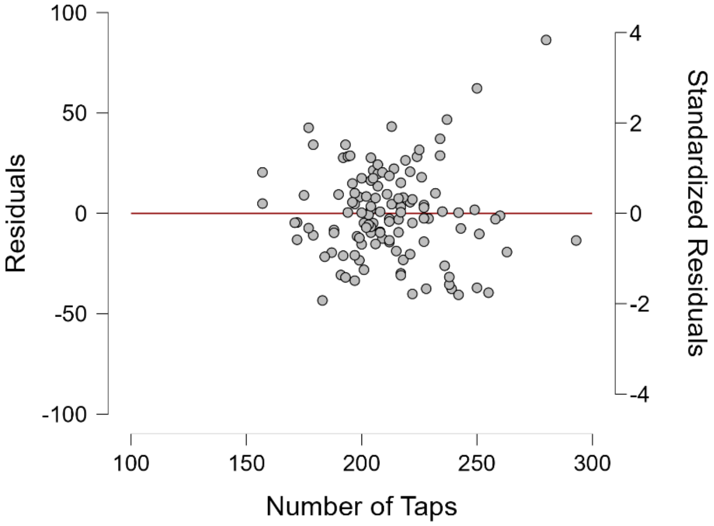

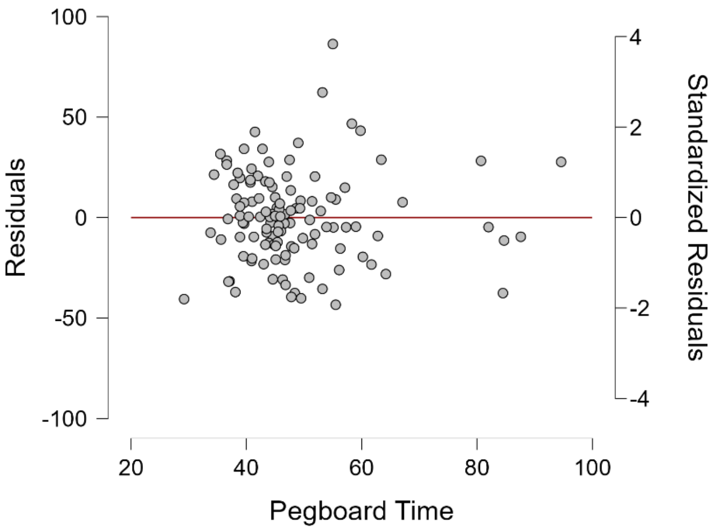

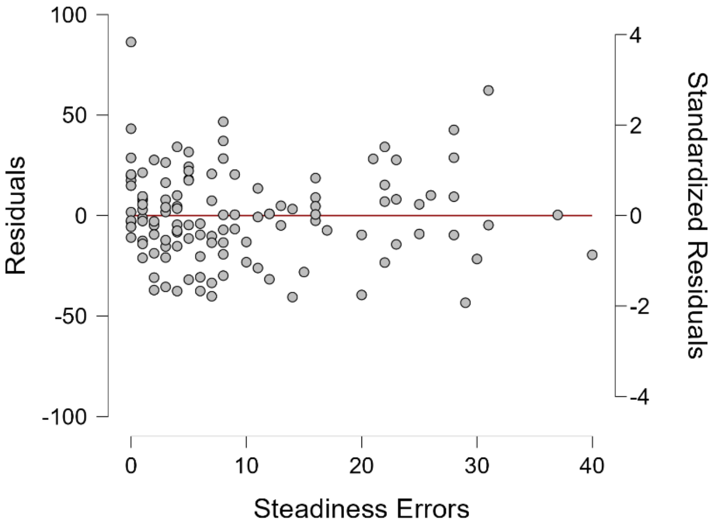

2. Multiple Regression with FAIR L as criterion variable

2.1 Normality

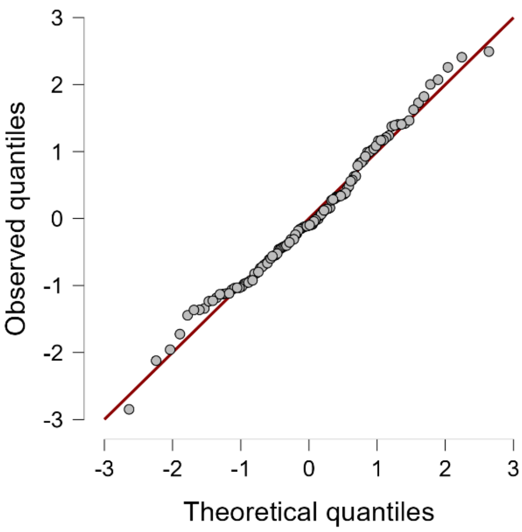

2.2 Homoscedasticity

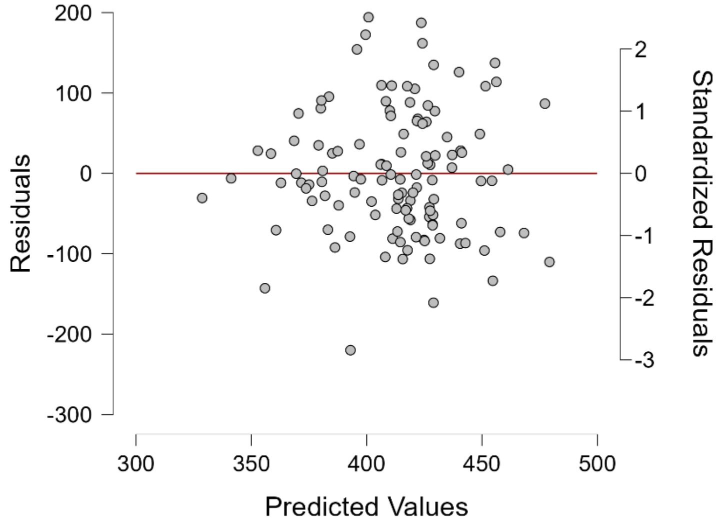

2.3 Linearity

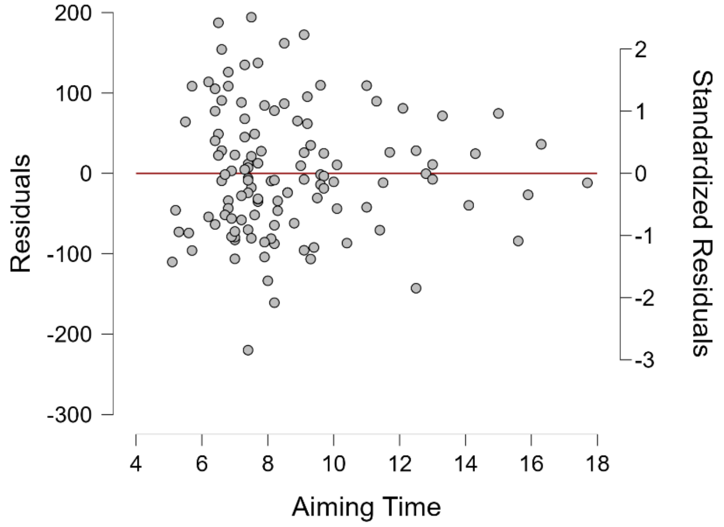

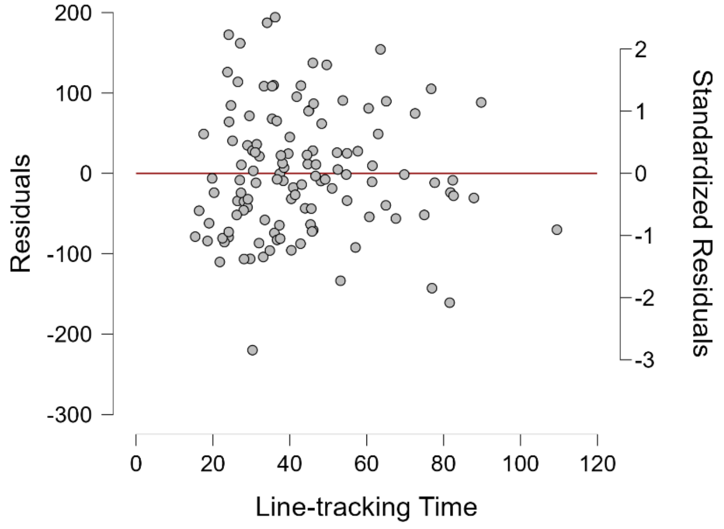

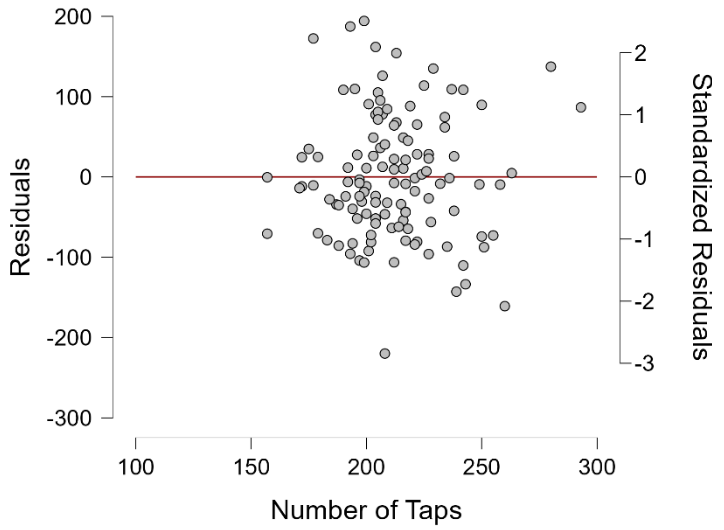
Residuals
Standardized Residuals
Number of Taps
200
100
0
-100
-200
-300
100
150
200
250
300
2
1
0
-1
-2
-3

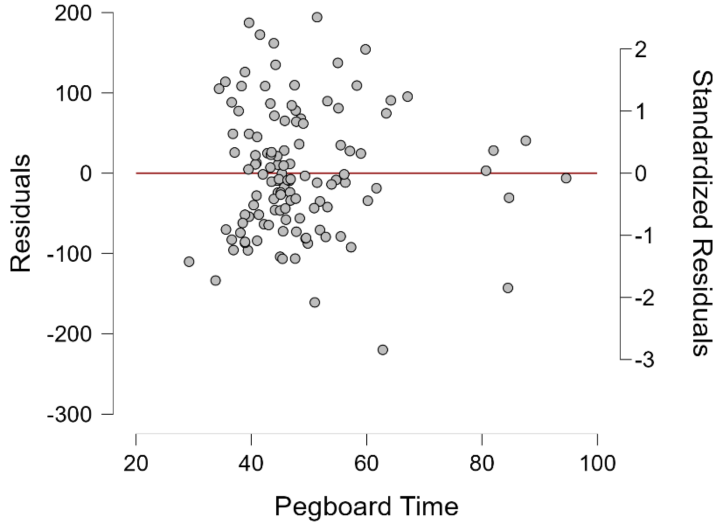
Residuals
Standardized Residuals
Pegboard Time
200
100
0
-100
-200
-300
20
40
60
80
100
2
1
0
-1
-2
-3

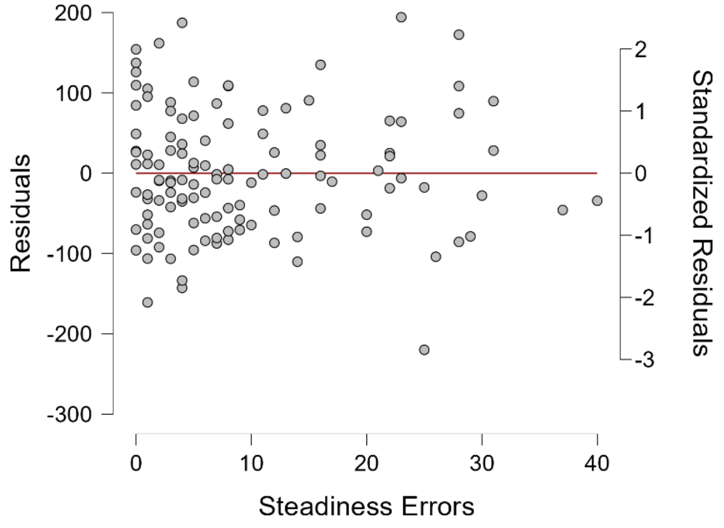
Residuals
Standardized Residuals
Steadiness Errors
200
100
0
-100
-200
-300
0
10
20
30
40
2
1
0
-1
-2
-3

3. Multiple Regression with d2 error percentage as criterion variable

3.1 Normality

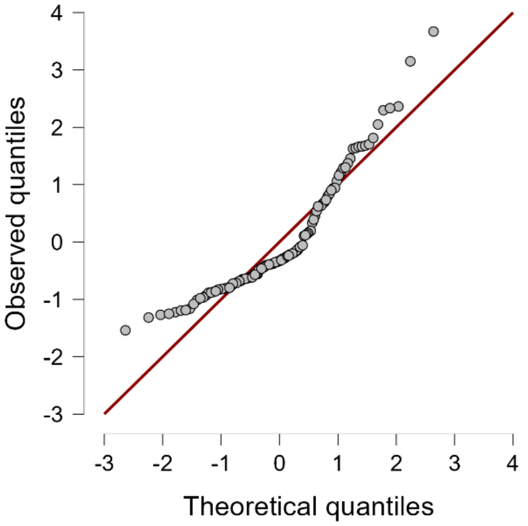

3.2 Homoscedasticity

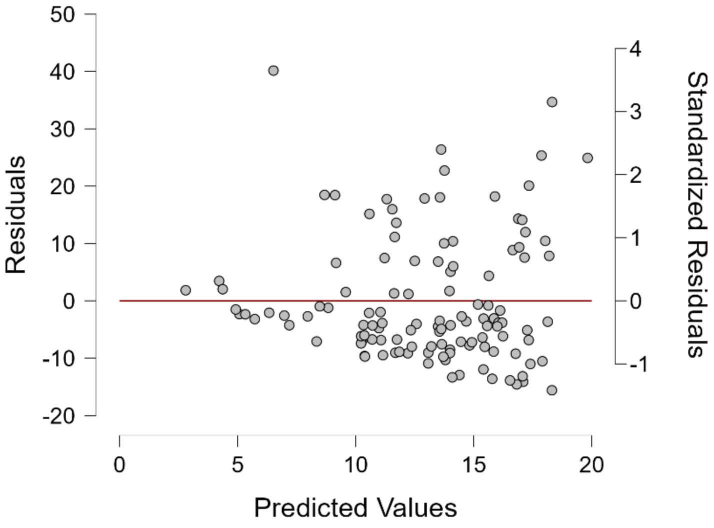

3.3 Linearity

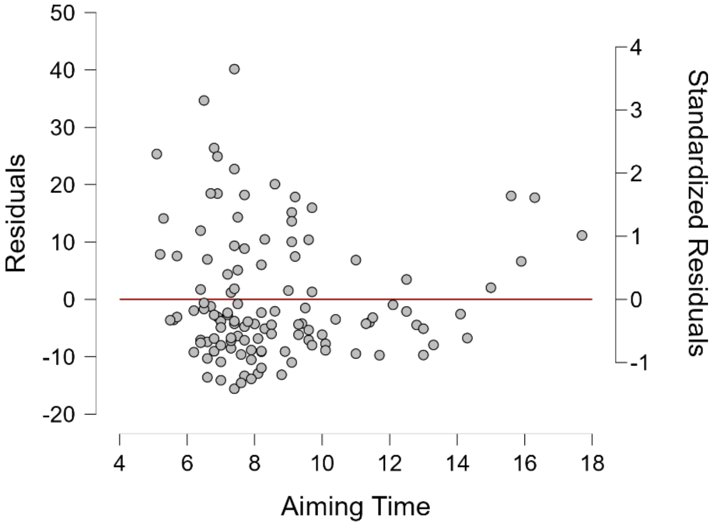
Residuals
Standardized Residuals
Aiming Time

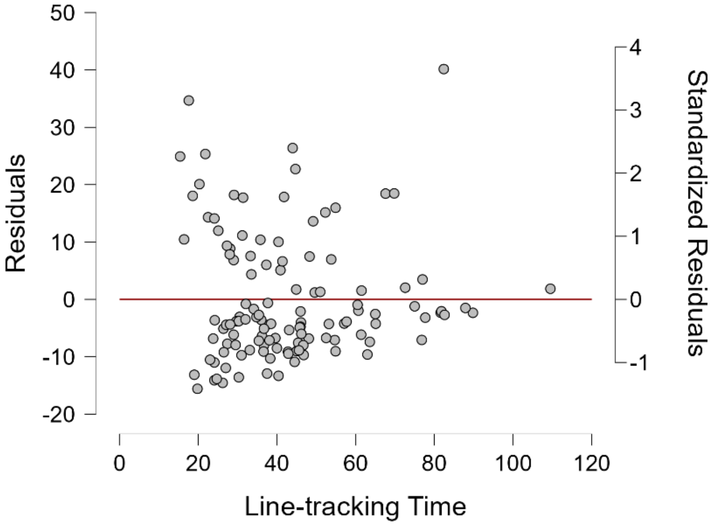
Residuals
Standardized Residuals
Line-tracking Time

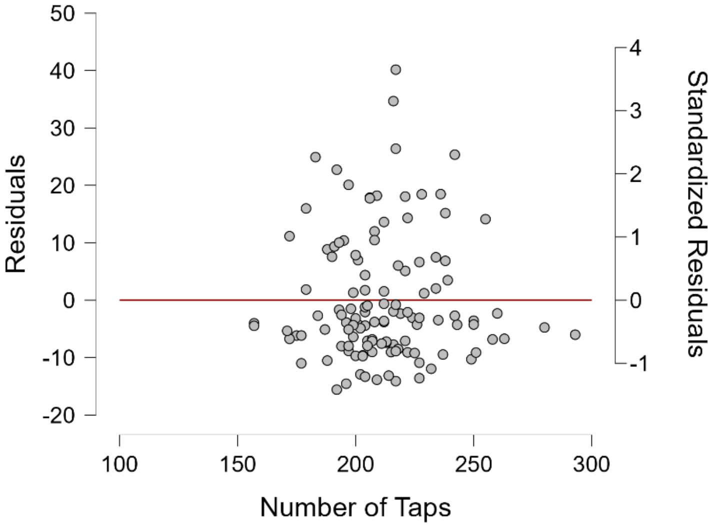
Residuals
Standardized Residuals
Number of Taps

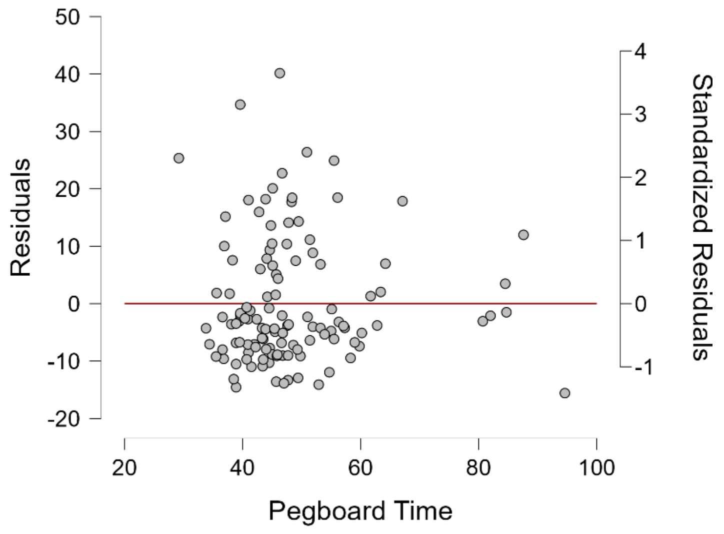

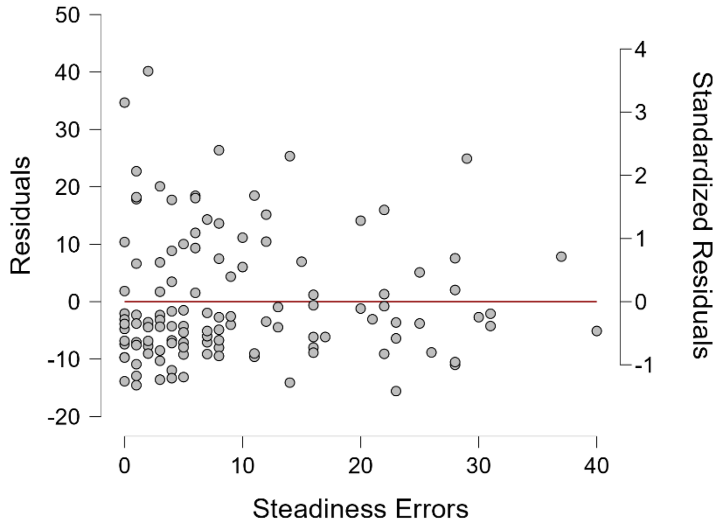

4. Multiple Regression with FAIR Q as criterion variable

4.1 Normality

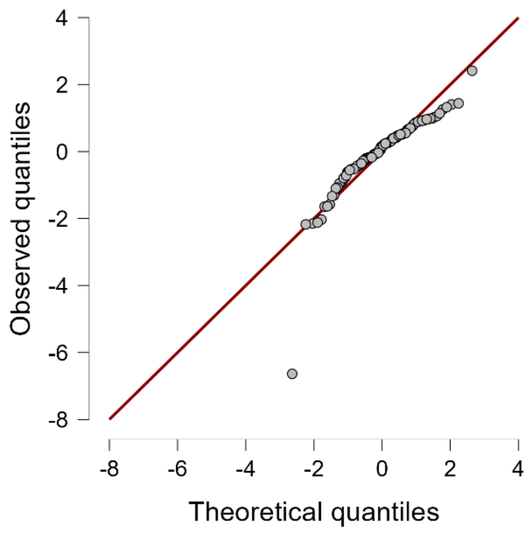

4.2 Homoscedasticity

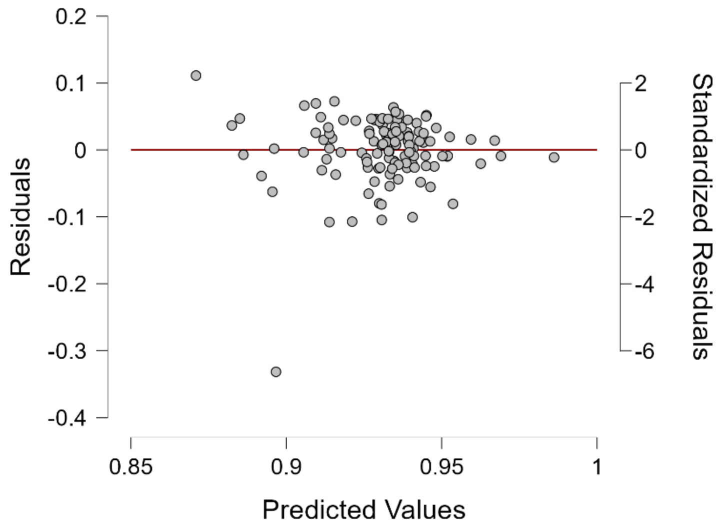

4.3 Linearity

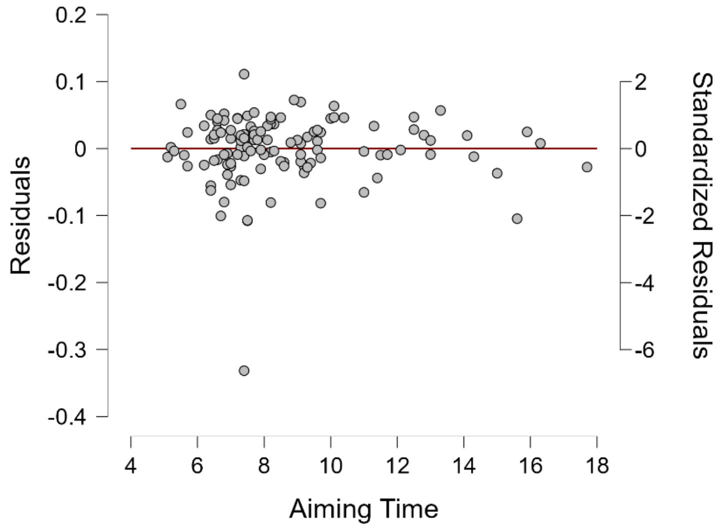

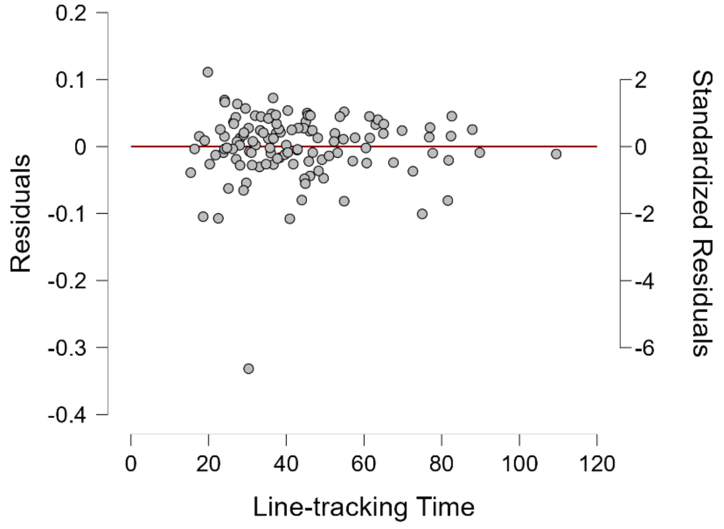

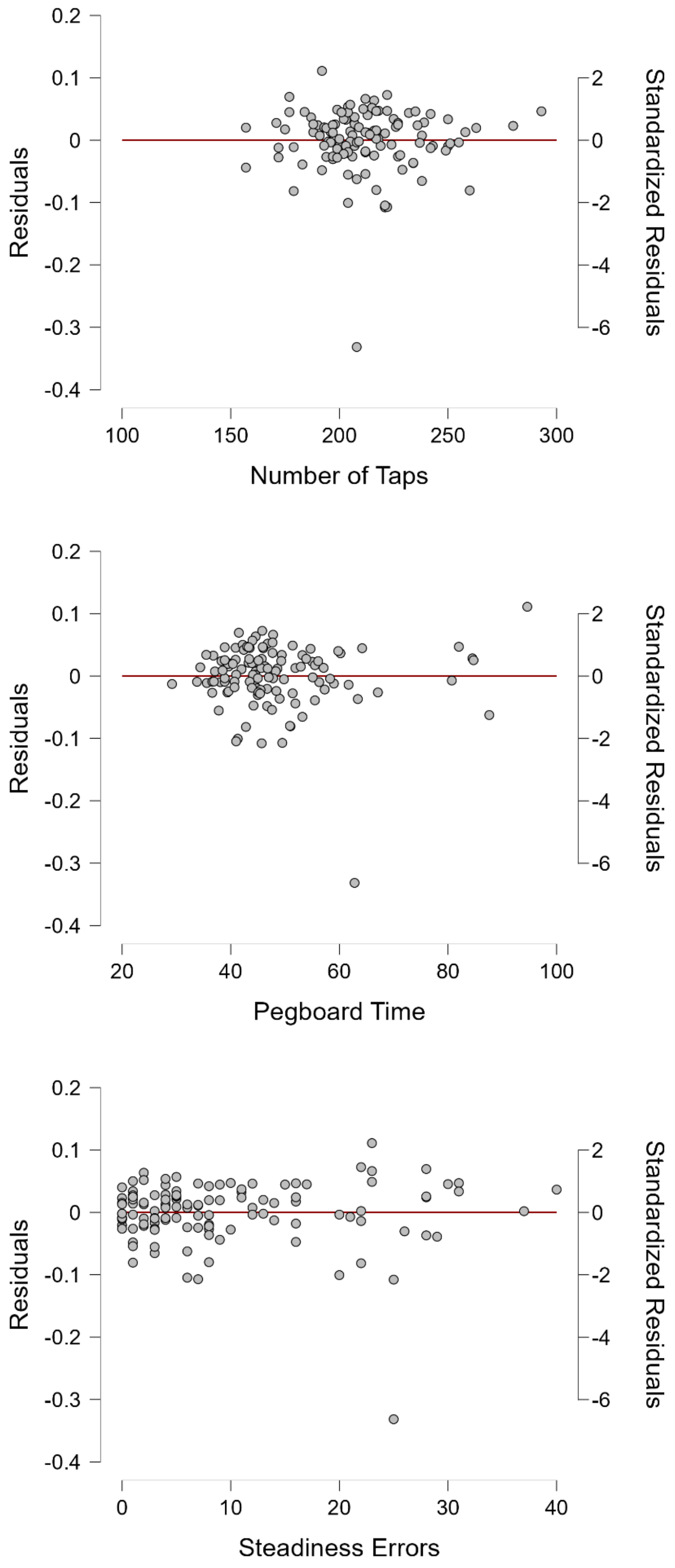
0.2
0.1
0
-0.1
-0.2
-0.3
-0.4
Residuals
2
0
-2
-4
-6
Standardized Residuals
100
150
200
250
300
Number of Taps
0.2
0.1
0
-0.1
-0.2
-0.3
-0.4
Residuals
2
0
-2
-4
-6
Standardized Residuals
20
40
60
80
100
Pegboard Time
0.2
0.1
0
-0.1
-0.2
-0.3
-0.4
Residuals
2
0
-2
-4
-6
Standardized Residuals
0
10
20
30
40
Steadiness Errors

5. Multiple Regression with BIS measure for d2-R as criterion variable

5.1 Normality

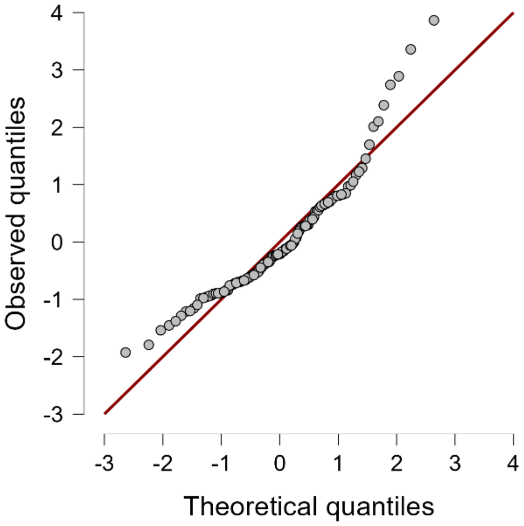

5.2 Homoscedasticity

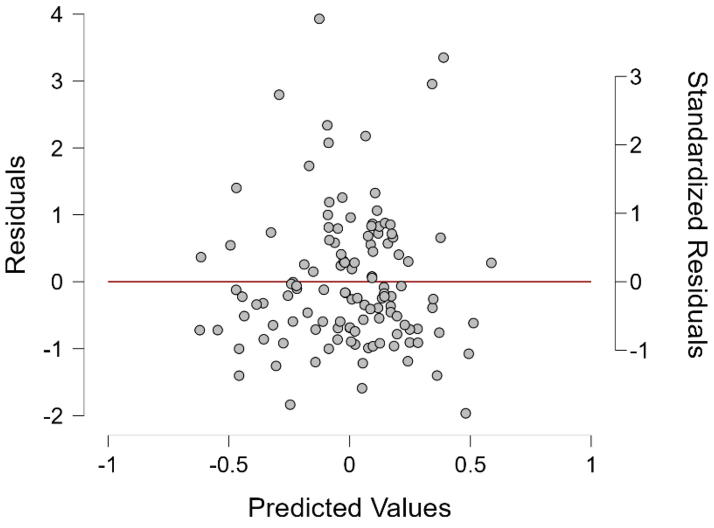

5.3 Linearity

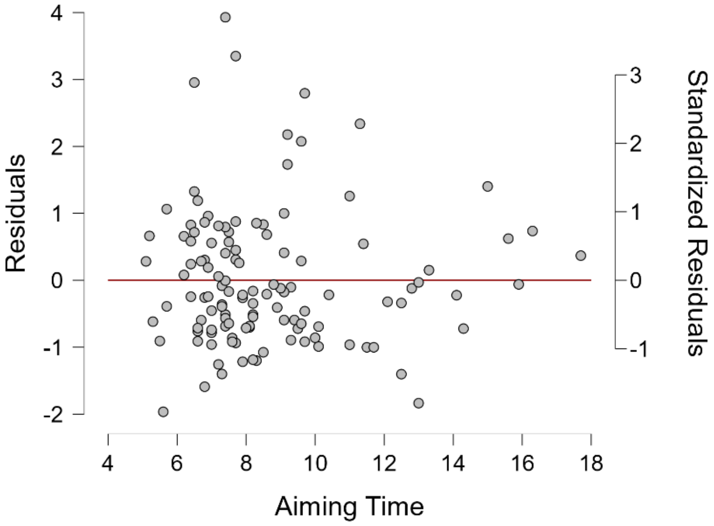
Residuals
Standardized Residuals
Aiming Time
4
3
2
1
0
-1
-2
4 6 8 10 12 14 16 18

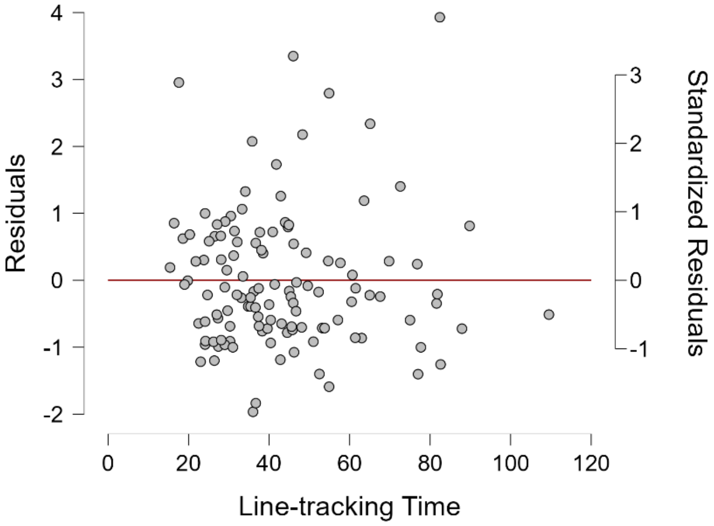
Residuals
Standardized Residuals
Line-tracking Time
0 20 40 60 80 100 120

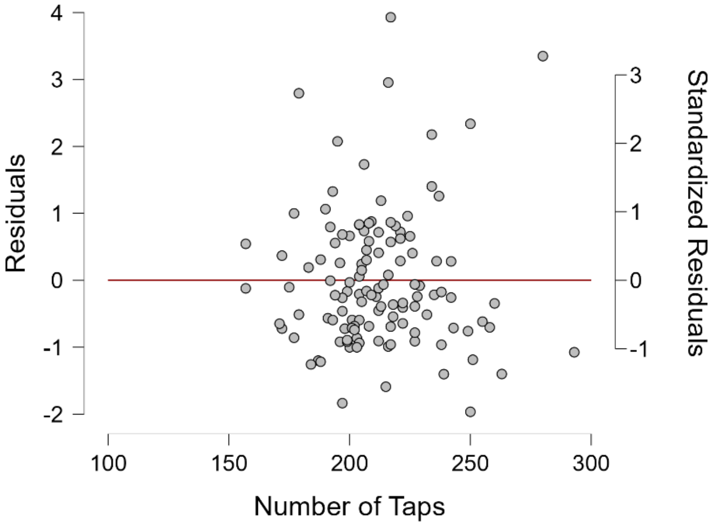
Residuals
Standardized Residuals
Number of Taps
100 150 200 250 300

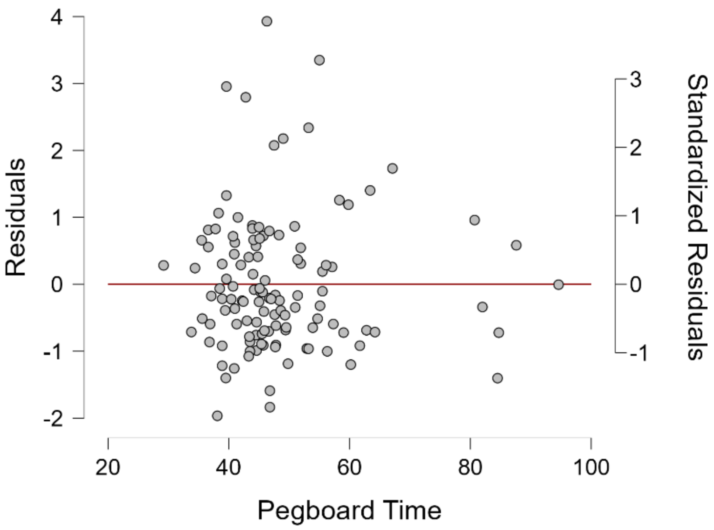

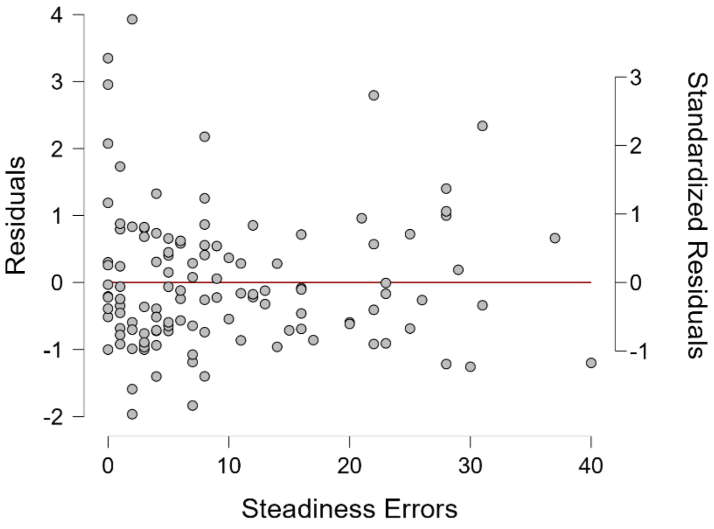

6. Multiple Regression with BIS measure for FAIR2 as criterion variable

6.1 Normality

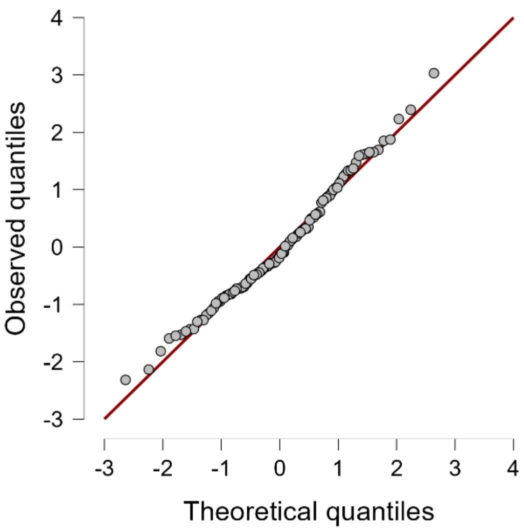

6.2 Homoscedasticity

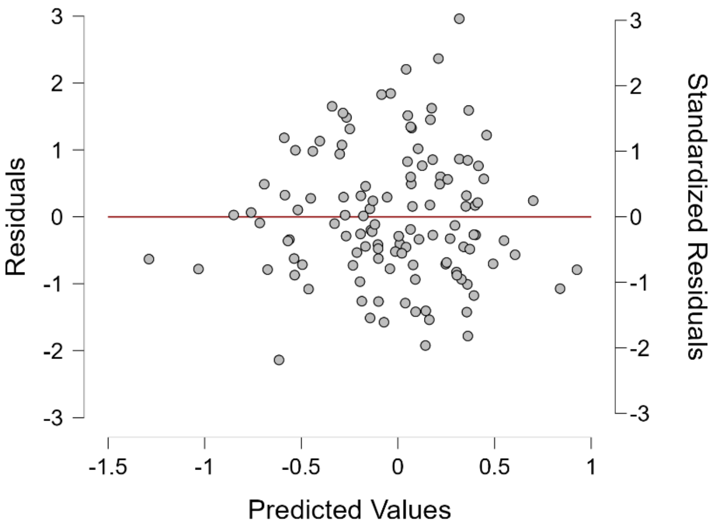

6.3 Linearity

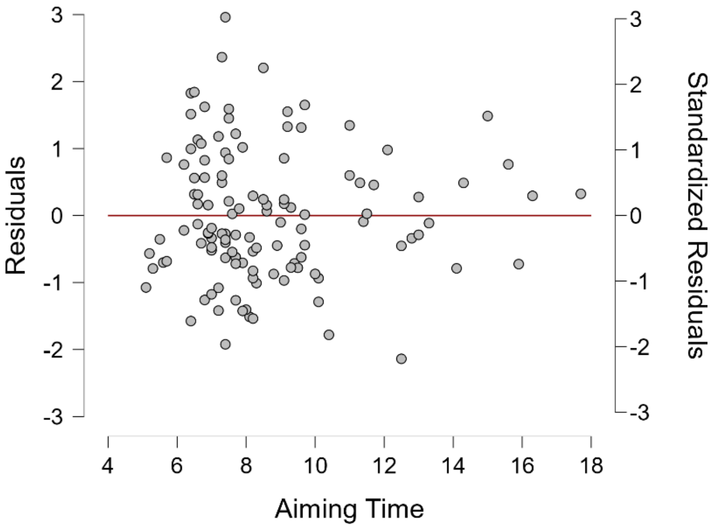

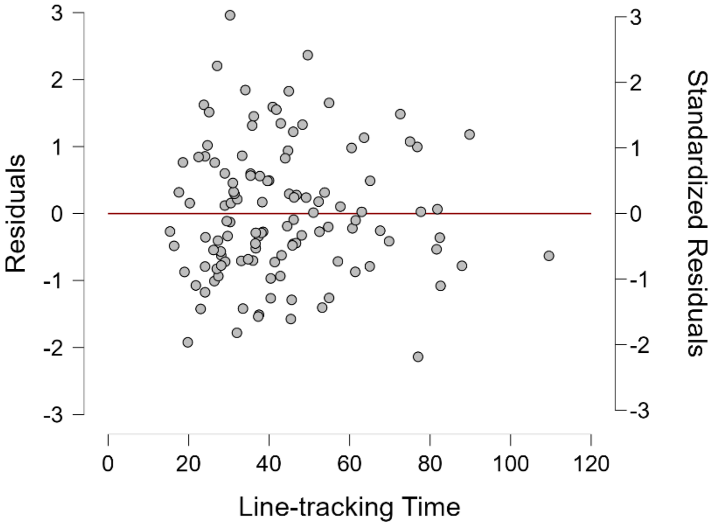

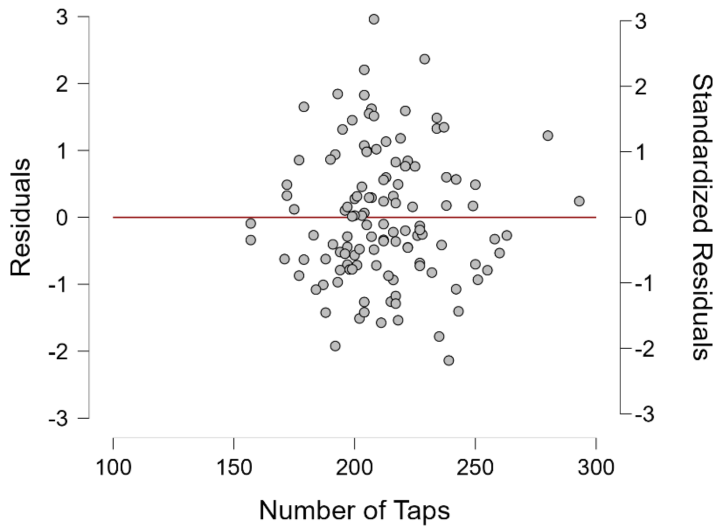

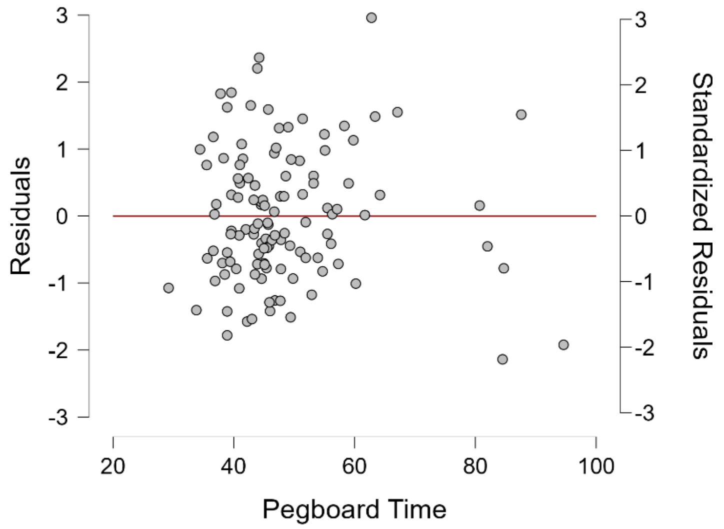

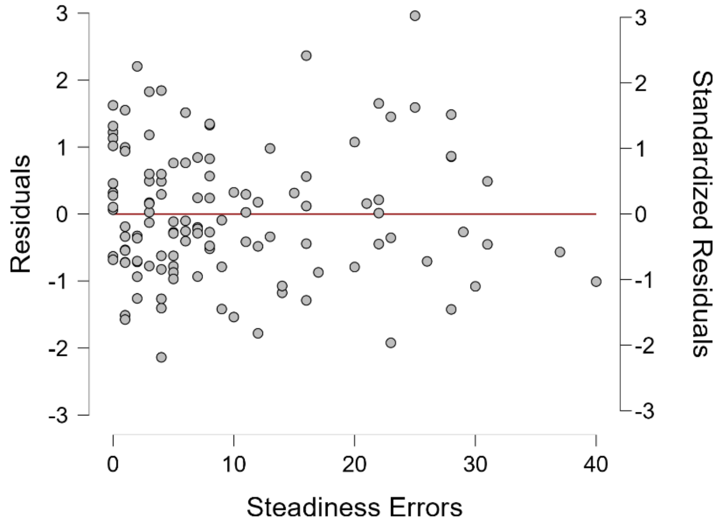

All figures were produced with JASP version 0.19.3.
